# Utilisation of semiconductor sequencing for the detection of predictive biomarkers in glioblastoma

**DOI:** 10.1371/journal.pone.0245817

**Published:** 2022-03-24

**Authors:** Gareth Williams, Alexander Llewelyn, Robert Thatcher, Keeda-Marie Hardisty, Marco Loddo

**Affiliations:** Oncologica UK Ltd, Cambridge, United Kingdom; The Jackson Laboratory for Genomic Medicine, UNITED STATES

## Abstract

The standard treatment for glioblastoma involves a combination of surgery, radiation and chemotherapy but have limited impact on survival. The exponential increase in targeted agents directed at pivotal oncogenic pathways now provide new therapeutic opportunities for this tumour type. However, lack of comprehensive precision oncology testing at diagnosis means such therapeutic opportunities are potentially overlooked. To investigate the role of semiconductor sequencing for detection of predictive biomarkers in routine glioblastoma samples we have undertaken analysis of test trending data generated by a clinically validated next generation sequencing platform designed to capture actionable genomic variants distributed across 505 genes. Analysis was performed across a cohort of 55 glioblastoma patients. Analysis of trending data has revealed a complex and rich actionable mutational landscape in which 166 actionable mutations were detected across 36 genes linked to 17 off label targeted therapy protocols and 111 clinical trials. The majority of patients harboured three or more actionable mutations affecting key cancer related regulatory networks including the PI3K/AKT/MTOR and RAS/RAF/MEK/MAPK signalling pathways, DNA-damage repair pathways and cell cycle checkpoints. Linkage with immunotherapy and PARP inhibitors was identified in 44% of glioblastoma patients as a consequence of alterations in DNA-damage repair genes. Taken together our data indicates that precision oncology testing utilising semiconductor sequencing can be used to identify a broad therapeutic armamentarium of targeted therapies and immunotherapies that can be potentially employed for the improved clinical management of glioblastoma patients.

## Introduction

Gliomas are the most common tumours of the primary central nervous system, and of these, over a half of cases represent glioblastoma (GBM) at diagnosis. In the US approximately 70,000 primary CNS tumours are diagnosed, GBM being the most frequent high-grade glioma, with an incidence of 3–4/100,000 [1]. This has the most malignant phenotype of primary brain cancers and is associated with a poor prognosis with a median survival of around 14–18 months [2]. Its inherently disabling effects on patients, often to prevent independent function, result in a significant burden on health-care systems which is out of proportion with its incidence. The present conventional treatment protocols (surgery, radiation and the alkylating chemotherapy agent temozolomide) have limited impact in improving patient survival [3–5]. A range of new approaches have been developed to broaden the potential therapies available to glioblastoma patients, which include the use of Gamma Knife radiosurgery, hyperthermia and the development of new pharmacological tools such as targeted therapies and immunotherapies [6].

The last decade has seen major advances in dissecting the aberrant molecular pathways that contribute to glioblastoma development. Comprehensive genetic screens have shown that genetic alterations in glioblastoma are distributed across the entire genome, resulting in the dysregulation of many critical signalling pathways such as the RB and p53 pathways and the receptor tyrosine kinase/Ras/phosphoinositide 3-kinase signalling pathways [7, 8]. There are also errors in DNA replication, DNA repair, chromosomal segregation and disruption of cell cycle checkpoints which all contribute to uncontrolled cell proliferation [9–11]. The wide range of molecular events in this tumour type therefore provides new potential therapeutic opportunities using the new generation of targeted anti-cancer agents including small molecule inhibitors and humanized monoclonal antibodies which have been specifically developed against these molecular targets.

The increasing use of targeted agents offers the great advantage of increased specificity and reduced toxicity when compared with conventional chemotherapy [12]. Meta-analysis in diverse tumour types has shown that a personalized strategy of treatment is an independent predictor of better outcomes and fewer toxic deaths when compared with chemotherapy [13]. However, it is critical that targeted agents are precisely matched to the correct patients based on their tumour mutation profile. When prescribed in the absence of tumour molecular profiling, targeted agents actually have poorer outcomes when compared with cytotoxics [13]. Identification of the most appropriate targeted therapies for cancer patients is currently a challenge because molecular profiling tests used in routine clinical practice are limited in their coverage and do not provide direct evidence-based linkage with therapy. Moreover, many sequencing platforms are unable to operate efficiently when applied to small clinical biopsy samples which have undergone formalin fixation resulting in low DNA/RNA yields with low integrity and quality [14]. At the present time routine molecular testing with regard to glioblastoma is restricted to a limited number of biomarkers such as IDH mutation status, MGMT promoter methylation and 1p19q co-deletion FISH analysis [15]. These biomarkers provide prognostic information and weak prediction of chemotherapy response. However, the new era of targeted next-generation sequencing (NGS) technologies, optimized for nucleic acid templates extracted from routine formalin fixed, paraffin embedded tumour samples (PWET), now provides the opportunity for comprehensive predictive testing in solid tumours.

To investigate the potential role of clinically directed semiconductor targeted sequencing in solid tumours we have established a clinically-validated next generation sequencing (NGS) platform optimized for the analysis of PWET clinical biopsy samples. This platform enables capture of 764 lead anti-cancer targeted agents/combinations and immunotherapy opportunities via analysis of actionable variants distributed across 505 genes. All actionable variants analysed are linked to targeted therapies either on-market FDA and EMA approved, carrying ESMO and NCCN guideline references or currently in clinical trials, phases I-IV, worldwide.

Here we have conducted a retrospective analysis of precision oncology profiling test trending data relating to a cohort of 55 glioblastoma patients who underwent testing following failure of first line treatment protocols. This analysis has shown that semiconductor sequencing can be applied robustly to routine processed glioblastoma samples enabling detection of a broad range of actionable mutations and their cognate therapies. The high frequency of actionable mutations detected in this tumour type demonstrates the broad range of potential therapeutic opportunities that are now available to glioblastoma patients following clinically directed precision oncology profiling.

## Materials and methods

### Patient demographics

A retrospective analysis was performed on the trending data generated as part of routine comprehensive precision oncology NGS testing for solid tumours and collected in compliance with ISO15189:2012 requirements for monitoring of quality indicators. Anonymized and coded patient data was collected between January 2018 and July 2019. A database search identified 55 cases of glioblastoma which were included in the analysis. The patient demographics including prognostic biomarker status for this cohort are shown in S1 Table. The pathological diagnosis was established by the pathologist at the treating hospital and independently verified by a state registered pathologist based at Oncologica.

### Comprehensive NGS genomic profiling

The NGS platform utilized for clinical testing is validated for clinical use and accredited by CLIA (ID 99D2170813) and by UKAS (9376) in compliance with ISO15189:2012 and following the guidelines published by the Association for Molecular Pathology and College of American Pathologists and IQN-Path ASBL [16, 17]. The performance characteristics are shown in S2 Table. The NGS platform targets 505 genes and detects actionable genetic variants linked to 764 anti-cancer targeted therapies/therapy combinations. This includes analysis of 51 driver and 349 partner genes for detection of 867 actionable fusion genes. Genomic regions selected for analysis of clinically relevant actionable variants are shown in S3 Table.

### DNA and RNA extraction, library preparation and sequencing

DNA and RNA was extracted from PWET curls cut at 10μm or from 5μm sections mounted onto unstained glass slides using the RecoverAll™ extraction kit (Ambion, Cat: A26069). RNA samples were diluted to 5ng/μl and reverse transcribed to cDNA in a 96 well plate using the Superscript Vilo cDNA synthesis kit (CAT 11754250). Library construction, template preparation, template enrichment and sequencing were performed using Ion AmpliseqTM library 2.0 (Cat: 4480441) and the Ion 540™ OT2 kit (Cat: A27753) according to the manufacturer’s instructions. Sequencing was performed using the Ion S5 system 20 (Cat: A27212) utilising Ion 540™ chips (Cat: A27766).

### Quality control metrics

Sequencing runs were quality controlled using the following parameters according to the manufacturer’s instructions (Ion Ampliseq library 2.0): chip loading >60% with >45 million reads observed, enrichment 98–100%, polyclonal percentage <55%, low quality <26%, usable reads > 30% and aligned bases were ≥80%, unaligned bases were <20%, mean raw accuracy was >99% and overall read length between 100-115bp for DNA and RNA. Individual DNA sample metrics were evaluated using the following parameters: number of mapped reads >4.5 million, percent reads on target >90%, average base coverage depth >1200, uniformity of amplicon (base) coverage >90%, amplicons less than 90% strand bias with >80% of amplicons reading end to end, on-target reads >85% and target base coverage at 1x, 20x, 100x and 500x >90% (S4 Table).

### Data analysis

Sequence alignment and variant calling was performed on The Torrent Suite™ Software (5.8.0). Alignment in Torrent Suite™ Software was performed using TMAP. The output BAM file was uploaded via the Ion Reporter Uploader plugin (5.8.32–1) to The Ion Reporter™ Software (5.10.1.0). Gene fusions were reported when occurring in >40 counts and meeting the threshold of assay specific internal RNA quality control with a sensitivity of 99% and PPV of 99%. Six internal expression quality controls were spiked into each sample to monitor assay performance with an acceptance cut-off of >15 reads in 5 out 6 controls (Ion Reporter™ 5.10.1.0; default fusion view [5.10]). Hotspot variants with >10% alternate allele reads were classified as ‘detected’ with an assay sensitivity and positive predictive value (PPV) of 99% (S2 Table). For copy number variants (CNV), amplifications of CN >6 with the 5% confidence value of ≥4 after normalization and deletions with 95% CI ≤1 were classified as present when the tumour content was >50% with a sensitivity of 80% and PPV of 100% (S2 Table). Variants meeting the previously defined criteria were then assessed for pathogenicity and linkage to cognate therapies using several resources including USCS, COSMIC, ClinVar databases, PhyloP, SIFT, Grantham, ExAC, DrugBank, NCI-MATCH, PFAM, DGV, MAF, GlobalData, PolyPhen in silico tools and IGV visualisation software. The results of variant annotation were organized hierarchically by gene, alteration, indication and level of evidence in relation to clinical actionability following the joint recommendation of the association of the AMP/ASCO/CAP (16) and the ESMO Scale for Clinical Actionability of Molecular Targets [18] including FDA/EMA approved therapies, guideline references by ESMO/NCCN and clinical trials (Phases I-IV) worldwide.

### Immunohistochemistry for PD-L1

An immunohistochemistry assay for the detection of PD-L1 was validated for clinical use and accredited by CLIA and by UKAS (9376) in compliance with ISO15189:2012. PD-L1 rabbit monoclonal antibody (clone E1L3N) was obtained from Cell Signalling (Cat: 13684S). Histological sections from a representative PWET block for each case were cut at 3Δm thickness and mounted on Super Frost glass slides (Leica, Cat: 10149870). Section deparaffinization, antigen retrieval and immunohistochemical labelling were performed using the Bond III Autostainer and Bond Polymer Refine Detection Kit (Leica, Cat: DS8900) according to the manufacturer’s instructions. Primary antibody was applied for 20 minutes at 1/400 dilution. Assessment of PD-L1 immunostaining was performed by a qualified histopathologist in accordance with PD-L1 clinical reporting guidelines [19].

### Statistical analysis

The 2 tailed t-test assuming equal variances was used to compare the mean number of mutations across variant type using the 95% confidence interval and assessed as statistically significant at the 5% level. All tests including means and medians were calculated using Microsoft Excel 2010.

### Ethical statement

The research conducted in this study was limited to secondary use of information previously collected in the course of normal care (without an intention to use it for research at the time of collection) and therefore does not require REC review. The patients and service users were not identifiable to the research team carrying out trend data analysis. This is also in accordance with guidance issued by the National Research Ethics Service, Health Research Authority, NHS and follows the tenants of the Declaration of Helsinki.

## Results

**Fig 1:**
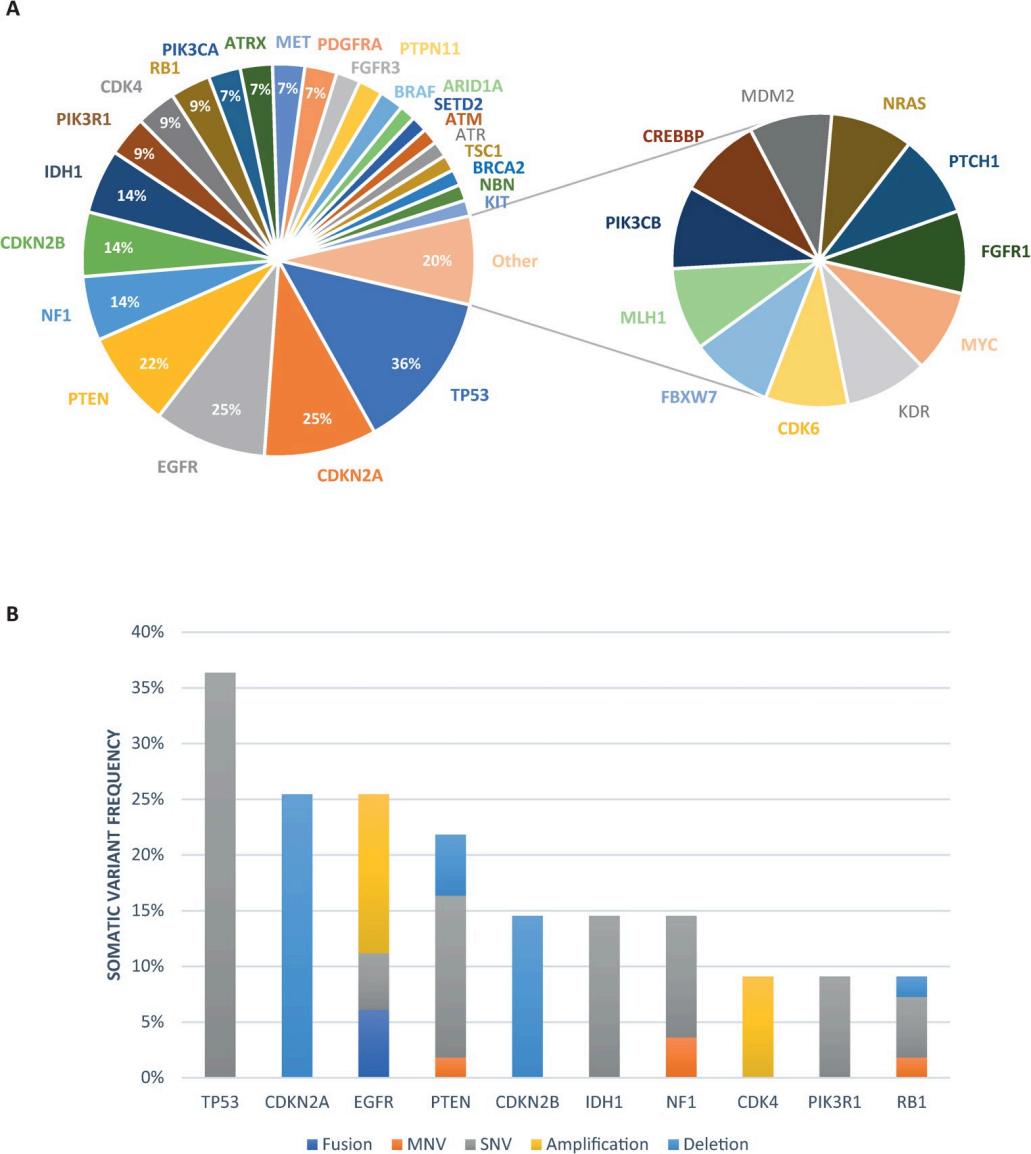
The actionable genomic landscape in glioblastoma. A) Pie chart showing frequency of altered genes in glioblastoma (n = 55). Segments not showing percentages have a frequency of <6%. B) Bar chart showing the ten most frequently genetically altered genes including variant type.

**Fig 2:**
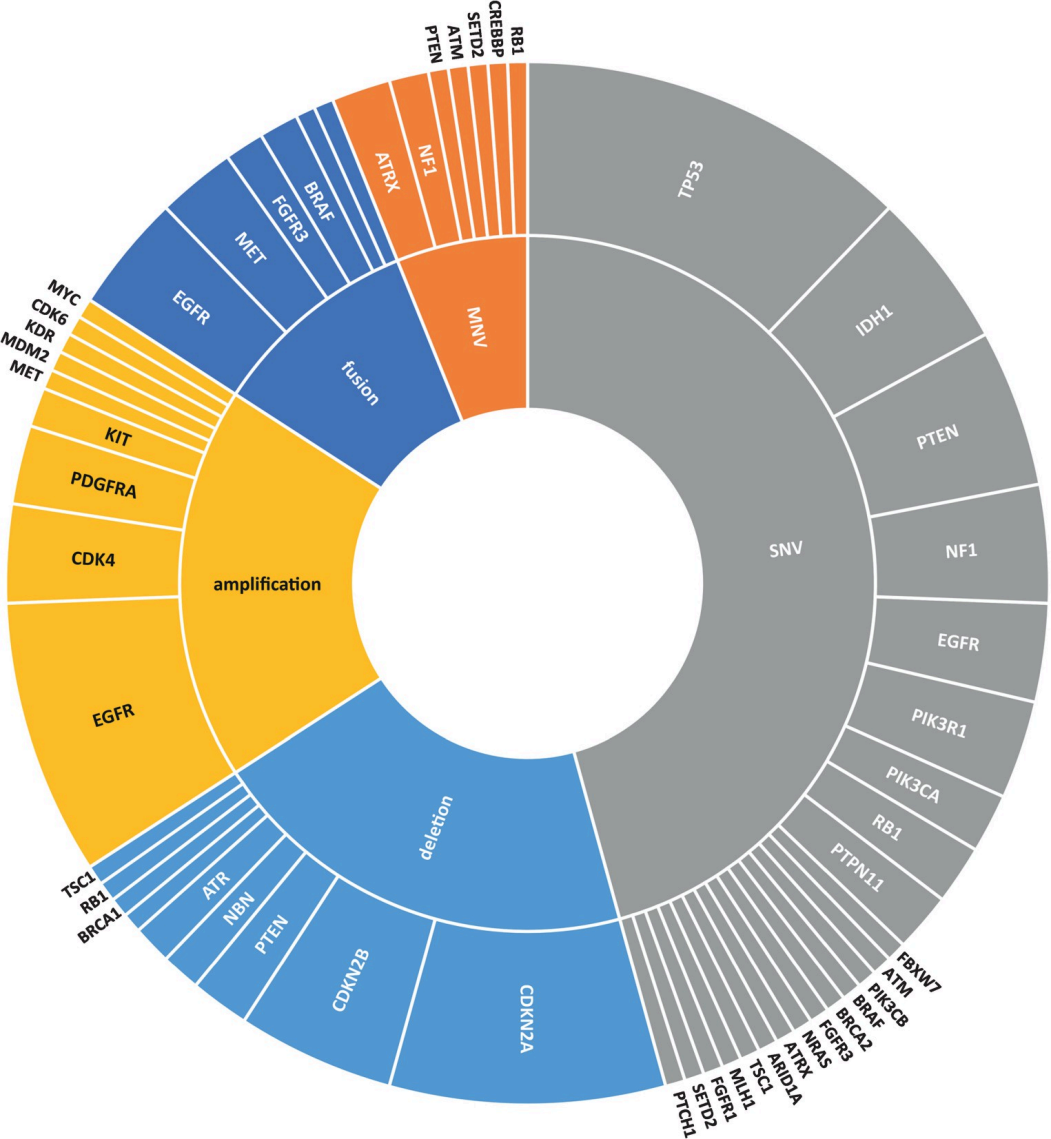
Frequency of actionable genetic alterations by variant type (inner ring) and by gene (outer ring) in all variants detected (n = 164).

**Fig 3:**
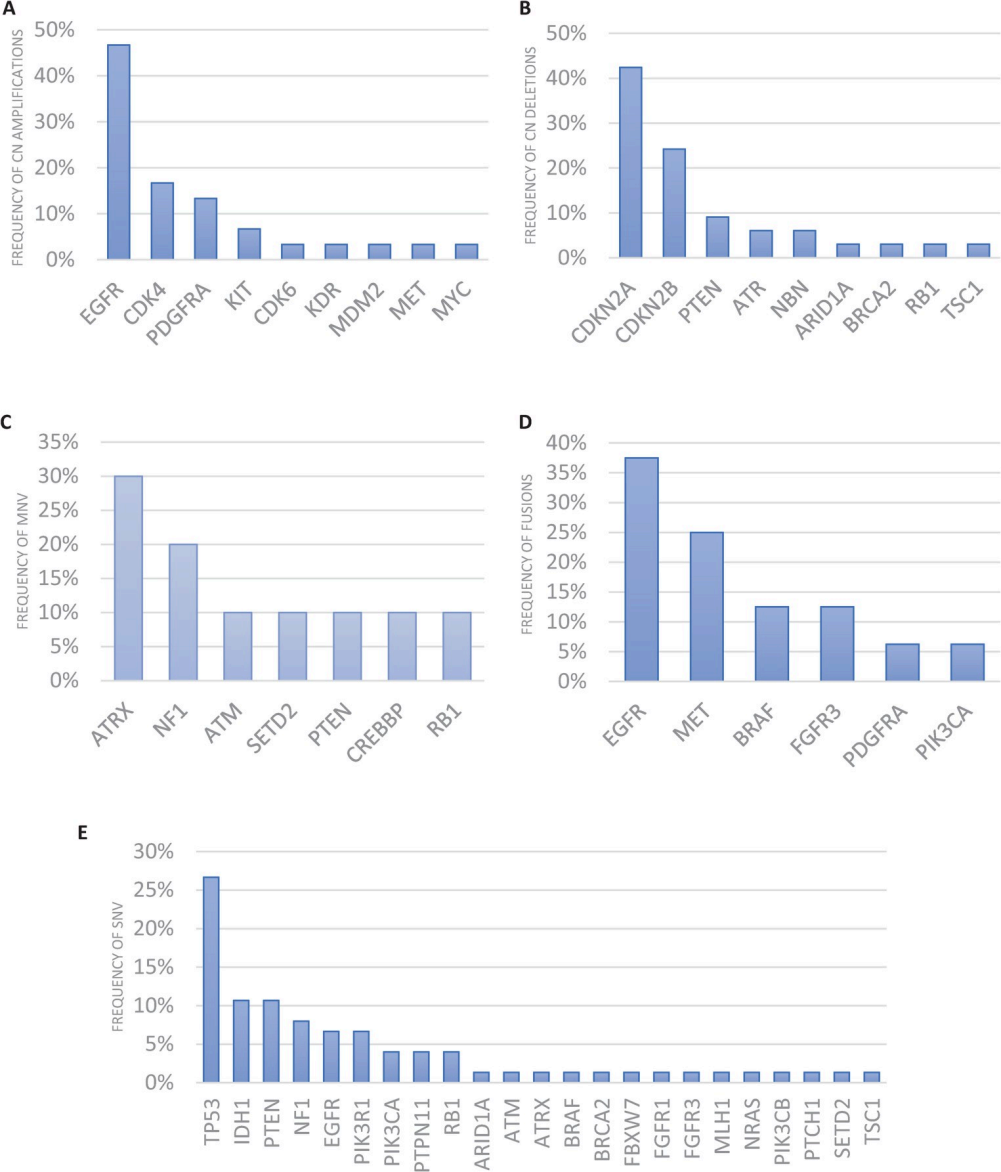
Frequency of altered genes in each variant type. A) Copy number amplifications; B) Copy number deletions; C) Multiple nucleotide variants (MNV); D) gene fusions and E) Single nucleotide variants (SNV).

Actionable somatic variants were identified in all glioblastoma samples tested and more than one variant was detected in 45 out of the 55 samples (82%). Across all 55 glioblastoma samples, one hundred and sixty-six actionable mutations were detected in 36 genes (Fig 1A, S1 Fig and S5–S7 Tables) with TP53, CDKN2A, EGFR, PTEN, CDKN2B, NF1, CDK4, IDH1, PIK3R1 and RB1 being the ten most frequently mutated (Fig 1B). The median number of actionable variants detected per sample was 3 and ranged from 1–9 (S2 Fig). Aberration of cancer genes occurs by a range of mechanisms including SNVs, gene amplification and deletion (collectively denoted CNVs), fusions and multiple nucleotide variants (MNVs) which include insertions, deletions and duplications. Their frequencies in relation to each cancer gene are shown in Figs 1B, 2 and 3. The mutational landscape is dominated by SNVs, detected in 69.1% of samples, and CNVs which occur at a similar frequency of 63.6% across all samples (S3 Fig).

Interestingly, a broad range of actionable fusion genes were identified in this cohort of glioblastomas including EGFR-SEPT, EFGR intragenic recombination, FGFR3-TACC3, PTPRZ1-MET, CAPZA2-MET, METex14 skipping, AGK-BRAF, EGFR Viii, TBLXR1-PIK3CA and FIP1L1-PDGFRA (Fig 3D and S7 Table). Although fusions are generally regarded as a rare event in solid tumours, with around a 3% frequency [20], here we identified druggable fusion genes in 23.6% of glioblastoma cases. Moreover, two of these fusions, TBL1XR1-PIK3CA and FIP1L1-PDGFRA, have not been previously reported in glioblastoma to our knowledge. The mean number of variants detected was significantly greater in samples containing fusions (3.92) versus absence of fusions (2.74) (p = 0.025) or deletions (3.81) versus absence of deletions (2.53) (p = 0.005).

There were no statistically significant differences in the frequency of non-DDR variants between DDR-positive and DDR-negative patients. However, at a gene level, mutations in TP53 were more common in DDR-positive patients (p = 0.030). Three patients were assessed for tumour mutation burden in the cohort, the two patients with low TMB (2Mut/Mb) had one and two actionable variants respectively including one DDR mutation (TP53 C238Y and PTEN I238T; EGFR amplification) compared with a patient with high TMB (107Mut/Mb) harbouring 9 actionable variants including 3 DDR mutations (CDKN2A Deletion, CDKN2B Deletion, IDH1 R132H, MLH1 Q301, NF1 Q236*, PIK3R1 N564D, PTEN G251D, TP53 R273C/H214L). Patients with IDH1 mutations (n = 8) had an average of 4.25 ± 0.77 actionable variants compared to 2.69 ± 0.23 variants for IDH wild type patients (p = 0.016). There was no difference in the frequency of actionable variants between primary and recurrent glioblastomas (3.02 v 3.0) but our cohort did not include matched samples.

A variant-level analysis of the data revealed four co-occurrence correlations with potential biological significance for variants with n > 5: p53 activators CDKN2A and CDKN2B (p = 0.0024), EGFR A289V and EGFR Amplification (p = 0.0031), IDH1 R132H and PIK3R1 N564D (p = 0.0071), and IDH1 R132H and TP53 R273C (p = 5.6 x 10^−5^). A gene-level analysis furthermore identified a correlation between mutations in IDH1 and ATRX (p = 0.0043). Moreover, there was no statistically significant difference in the number of variants found in MGMT-methylated (3.25 ± 1.09) vs MGMT-unmethylated cases (2.74 ± 0.55) except for IDH1 where all IDH1-mutated samples (n = 8) were MGMT-methylated (p = 0.0049 by Fisher’s exact test).

All patients harboured one or more actionable variants linked bioinformatically to a wide range of targeted therapies with either FDA, EMA, NCCN or ESMO labels in relation to “cancers of other type” (off-label), or alternatively investigational targeted therapies in clinical trials (Table 1). The actionable variants in this cohort of patients were linked to 17 off-label targeted therapy protocols with FDA, EMA, NCCN or ESMO labels and meeting the tier criteria IIC level of clinical significance as defined by the Joint Consensus Recommendation of the Association for Molecular Pathology, American Society of Clinical Oncology, and College of American Pathologists [16]. This cohort of patients was additionally linked to a total of 111 clinical trials (Table 1) through matching of actionable variants to their cognate targeted therapies. A number of the therapeutic actionable variants identified in our cohort, namely EGFR, BRAF and IDH1, also have evidence-based linkage under the AMP/ASCO guidelines as diagnostic and prognostic markers [16] (Table 1). Out of the potential 58 drug matches, 41 therapies show evidence of crossing the blood brain barrier (S8 Table).

**Table 1:** Actionable drug targets in glioblastoma.

GENOMIC ALTERATION	PATHWAY	RELEVANT CLASSES OF THERAPY	RELEVANT THERAPIES *APPROVAL IN OTHER CANCER TYPE **OFF-LABEL/CLINICAL TRIALS	CLINICAL TRIALS
BRAF V600E MUTATION *TIER 2D DIAGNOSTIC* *TIER 2C PROGNOSTIC*	• RAS/RAF/MEK/ MAPK	• BRAF inhibitors • MEK inhibitors • MAPK inhibitors	*Binimetinib + encorafenib *Cobimetinib + vemurafenib *Dabrafenib + trametinib *Dabrafenib *Trametinib *Vemurafenib *BRAF inhibitor + MEK inhibitor *Cetuximab + vemurafenib + chemotherapy *Panitumumab + vemurafenib + chemotherapy	NCT02639546; NCT02693535; NCT02925234; NCT03297606; NCT03520075; NCT03781219 NCT03118817; NCT02407509; NCT03051035; NCT03634982; NCT03284502; NCT02857270 NCT02607813
**NRAS Q61 MUTATION**	• RAS/RAF/MEK/ MAPK	• RAS/RAF inhibitors • MEK inhibitors	*Contraindications*: *cetuximab + chemotherapy *cetuximab *Panitumumab + Chemotherapy *Panitumumab	NCT02639546; NCT02079740; NCT03637491; NCT03520075; NCT03415126; NCT03118817 NCT02407509; NCT03051035; NCT03284502; NCT03634982; NCT02857270; NCT02607813
			e.g. **sorafenib e.g. **trametinib	
**PIK3CA MUTATION**	• PI3K/AKT/MTOR	• PI3K inhibitors • AKT inhibitors • mTOR inhibitors	*Alpelisib + fulvestrant	NCT02029001; NCT02688881; NCT03297606; NCT02576444; NCT03673787; NCT03006172 NCT03065062; NCT02389842
**BRCA2 MUTATION**	• DNA Repair: Homologous Repair	• PARP inhibitors • PARP inhibitor + immunotherapy	*Olaparib *Rucaparib	NCT03767075; NCT03565991; ACTRN12617001000392; NCT02029001; NCT02693535; NCT02873975; NCT02286687 NCT03718091; NCT03297606; NCT02576444; NCT02401347; NCT03330405; NCT03188965 NCT03061188; NCT02660034
			e.g. **olaparib, rucaparib, talazoparib e.g. **atezolizumab + talazoparib	
**MET AMPLIFICATION**	• RAS/RAF/MEK/ MAPK • PI3K/AKT/MTOR	• MET inhibitors	*capmatinib *crizotinib	NCT03138083; NCT02386826; NCT03297606; NCT02648724; NCT02977364; NCT02219711
**ATM MUTATION**	• DNA Repair	• PARP inhibitors • PARP inhibitor + immunotherapy	e.g. **olaparib, rucaparib, talazoparib e.g. **atezolizumab + talazoparib	NCT03565991; NCT02576444 ACTRN12617001000392; NCT03207347; NCT02029001; NCT02693535; NCT02925234 NCT02873975; NCT02286687; NCT02401347; NCT03718091; NCT03767075; NCT03297606 NCT03330405; NCT03188965; NCT03061188; NCT02660034
**NBN MUTATION**	• DNA Repair	• PARP inhibitors • PARP inhibitor + immunotherapy	e.g. **olaparib, rucaparib, talazoparib e.g. **atezolizumab + talazoparib	NCT03207347; NCT02286687; NCT02401347; NCT03718091; NCT03767075; NCT02576444 NCT02029001; NCT03297606; NCT02873975; NCT03061188; NCT02660034
**PTEN MUTATION**	• PI3K/AKT/MTOR	• PI3K inhibitors • AKT Inhibitors • MTOR inhibitors	e.g. **alpelisib, capivasertib, everolimus	NCT02576444; NCT02029001; NCT03207347; NCT02286687; NCT02401347; NCT03297606 NCT03673787; NCT02761694; NCT01884285; NCT03065062; NCT02389842
EGFR AMPLIFICATION *TIER 2C DIAGNOSTIC* *TIER 2C PROGNOSTIC*	• RAS/RAF/MEK/ MAPK • PI3K/AKT/MTOR	• EGFR inhibitors	e.g. **erlotinib, gefitinib, lapatinib, neratinib	NCT03618667; NCT01953926; NCT03231501; NCT02423525; NCT03603379; NCT02101905 NCT03297606; NCT03065387; NCT03319459 5209-CPK-1002, CTR20150792
**CHEK2 MUTATION**	• DNA Repair / Cell cycle pathway	• PARP Inhibitor • PARP inhibitor + immunotherapy	e.g. **olaparib, rucaparib, talazoparib e.g. **atezolizumab + talazoparib	NCT02576444; ACTRN12617001000392 NCT03207347; NCT02873975; NCT02401347; NCT03718091; NCT03767075; NCT03188965 NCT03061188; NCT02660034
**ATR MUTATION**	• DNA Repair	• PARP inhibitor • PARP inhibitor + immunotherapy	e.g. **olaparib, rucaparib, talazoparib e.g. **atezolizumab + talazoparib	ACTRN12617001000392; NCT03207347; NCT02873975; NCT02286687; NCT02401347 NCT03767075; NCT03188965; NCT03061188; NCT02660034
**PTEN DELETION**	• PI3K/AKT/MTOR	• PI3K inhibitors • AKT Inhibitors • MTOR inhibitors	e.g. **alpelisib, capivasertib, everolimus	NCT02029001; NCT02688881; NCT02286687; NCT03297606; NCT02576444; NCT03673787 NCT02761694; NCT03065062; NCT02389842
**TSC1 MUTATION**	• PI3K/AKT/MTOR	• PI3K inhibitors • AKT Inhibitors • MTOR inhibitors	e.g. **alpelisib, capivasertib, everolimus	NCT02029001; NCT02465060; NCT02693535; NCT03297606; NCT02576444; NCT03673787 NCT03065062; NCT02389842
**BRCA2 DELETION**	• DNA Repair: Homologous Repair	• PARP inhibitor • PARP inhibitor + immunotherapy	e.g. **olaparib, rucaparib, talazoparib e.g. **atezolizumab + talazoparib	ACTRN12617001000392; NCT02286687; NCT03297606; NCT02873975; NCT03330405 NCT03188965; NCT02660034
EGFR MUTATION *TIER 2C DIAGNOSTIC* *TIER 2C PROGNOSTIC*	• RAS/RAF/MEK/ MAPK • PI3K/AKT/MTOR	• EGFR inhibitors	*erlotinib, gefitinib, lapatinib, neratinib	NCT01953926; NCT02423525; NCT03297606; NCT02716116; NCT03065387; NCT03319459 5209-CPK-1002, CTR20150792
**FGFR1 MUTATION**	• RAS/RAF/MEK/ MAPK • PI3K/AKT/MTOR • JAK/STAT • PLC/PKC	• FGFR inhibitors	e.g. **erdafitinib, TAS-120	NCT02052778; NCT02465060; NCT02691793; NCT03297606; NCT03160833; NCT01948297 NCT03235570
**FGFR3 MUTATION**	• RAS/RAF/MEK/ MAPK • PI3K/AKT/MTOR • JAK/STAT • PLC/PKC	• FGFR inhibitors	e.g. **erdafitinib, TAS-120	NCT02052778; NCT02465060; NCT02691793; NCT03297606; NCT03160833; NCT01948297 NCT03235570
**FGFR3-TACC3 FUSION**	• RAS/RAF/MEK/ MAPK • PI3K/AKT/MTOR • JAK/STAT • PLC/PKC	• FGFR inhibitors	e.g. **erdafitinib, TAS-120	NCT02052778; NCT02465060; NCT02272998; NCT03297606; NCT03160833; NCT01948297 NCT03235570
**KIT AMPLIFICATION**	• RAS/RAF/MEK/ MAPK; • PI3K/AKT/MTOR	• KIT inhibitors	e.g. **imatinib, sunitinib	NCT02461849; NCT02029001; NCT02693535; NCT03297606; NCT02272998; NCT02219711 NCT02571036 (with PDGFRA amplification / mutation)
**NF1 MUTATION**	• RAS/RAF/MEK/ MAPK	• RAS inhibitors • RAF inhibitors • MEK inhibitors • MAPK inhibitors	e.g. **sorafenib e.g. **trametinib	NCT02639546; NCT03297606; NCT03520075; NCT03634982; NCT02407509; NCT02857270 NCT02607813
**AGK-BRAF FUSION**	• RAS/RAF/MEK/ MAPK	• RAS inhibitors • RAF inhibitors • MEK inhibitors • MAPK inhibitors	e.g. **sorafenib e.g. **trametinib	NCT02639546; NCT03843775; NCT03520075; NCT02857270; NCT02607813; NCT03634982
**ATM DELETION**	• DNA repair	• PARP inhibitor • PARP inhibitor + immunotherapy	e.g. **olaparib, rucaparib, talazoparib e.g. **atezolizumab + talazoparib	ACTRN12617001000392 NCT02693535; NCT02286687; NCT03330405; NCT03188965; NCT02660034
**MLH1 MUTATION**	• DNA Repair: Mismatch Repair	• Immunotherapy • PARP inhibitors	e.g. **pembrolizumab, nivolumab e.g. **olaparib, rucaparib, talazoparib	NCT02658279; NCT03767075; NCT02873975; NCT02286687; NCT03188965; NCT02660034
**PDGFRA AMPLIFICATION**	• RAS/RAF/MEK/ MAPK • PI3K/AKT/MTOR	• KIT inhibitors	e.g. **imatinib, sunitinib	NCT02626364; NCT02571036; NCT02029001; NCT03297606; NCT02272998; NCT02219711
**PIK3R1 MUTATION**	• PI3K/AKT/MTOR	• PI3K inhibitors • AKT Inhibitors • MTOR inhibitors	e.g. **alpelisib, capivasertib, everolimus	NCT02029001; NCT02576444; NCT03297606; NCT03673787; NCT03065062; NCT02389842
**PTPN11 MUTATION**	• RAS/RAF/MEK/ MAPK	• RAS inhibitors • RAF inhibitors • MEK inhibitors • MAPK inhibitors	e.g. **sorafenib e.g. **trametinib	NCT02639546; NCT03520075;NCT02407509; NCT03634982; NCT02857270; NCT02607813
**TSC1 DELETION**	• PI3K/AKT/MTOR	• PI3K inhibitors • AKT Inhibitors • MTOR inhibitors	e.g. **alpelisib, capivasertib, everolimus	NCT02029001; NCT03297606; NCT02576444; NCT03673787; NCT03065062; NCT02389842
EGFR VIII FUSION	• RAS/RAF/MEK/ MAPK • PI3K/AKT/MTOR	• EGFR inhibitors	e.g. **erlotinib, gefitinib, lapatinib, neratinib	NCT03296696; NCT02844062; NCT02423525; NCT03297606; NCT03319459
FIP1L1-PDGFRA FUSION	• RAS/RAF/MEK/ MAPK • PI3K/AKT / MTOR	• KIT inhibitors	e.g. **imatinib, sunitinib	NCT02029001; NCT03297606; NCT02272998; NCT02219711; NCT02571036 (with KIT amplification)
**ARID1A MUTATION**	• Chromatin remodelling • DNA Repair	• PARP inhibitors	e.g. **olaparib, rucaparib, talazoparib	NCT03207347; NCT02286687; NCT03718091; NCT03297424
**ATRX MUTATION**	• Chromatin remodelling • DNA Repair	• PARP inhibitors • PARP inhibitor + immunotherapy	e.g. **olaparib, rucaparib, talazoparib e.g. **atezolizumab + talazoparib	NCT03207347; NCT03767075; NCT03188965; NCT02660034
**FBXW7 MUTATION**	• PI3K/AKT/MTOR	• PI3K inhibitors • AKT Inhibitors • MTOR inhibitors • CDK4/CDK6 inhibitors	e.g. **alpelisib, capivasertib, everolimus e.g. **palbociclib, ribociclib	NCT02873975; NCT03718091; NCT03297606; NCT01037790
IDH1 R132H *TIER 1A DIAGNOSTIC* *TIER 1A PROGNOSTIC*	• DNA Repair • Gene expression regulation	• IDH1 inhibitors • PARP inhibitors • Immunotherapy	*ivosidenib e.g. **olaparib, rucaparib, talazoparib e.g. **pembrolizumab, nivolumab	NCT03212274; NCT03557359; NCT03207347; NCT03684811
**KDR AMPLIFICATION**	• RAS/RAF/MEK/ MAPK • PI3K/AKT/MTOR	• RAS inhibitors • RAF inhibitors • KIT Inhibitors	e.g. **sorafenib e.g. **imatinib, sunitinib	NCT02029001; NCT02693535; NCT03297606; NCT02219711
**SETD2 MUTATION**	• Histone modification • DNA Repair	• PARP inhibitor • PARP inhibitor + immunotherapy	e.g. **olaparib, rucaparib, talazoparib e.g. **atezolizumab + talazoparib	NCT03284385; NCT03767075; NCT03188965; NCT02660034
**CDK4 MUTATION**	• Cell cycle pathway	• CDK4/CDK6 inhibitors	e.g. ** palbociclib, ribociclib	NCT03207347; NCT01037790; NCT03297606
**CDKN2A DELETION**	• Cell cycle pathway	• CDK4/CDK6 inhibitors	e.g. ** palbociclib, ribociclib	NCT02693535; NCT03297606; NCT01037790
**EGFR-SEPT FUSION**	• RAS/RAF/MEK/ MAPK • PI3K/AKT/MTOR	• EGFR inhibitors	e.g. **erlotinib, gefitinib, lapatinib, neratinib	NCT02423525; NCT03297606; NCT03319459
**PTCH1 MUTATION**	• Other Growth Factor signalling: Patched / Smoothened	• SMO inhibitors	e.g. **vismodegib	NCT02091141; NCT02465060; NCT03297606
MET (EXON 14 SKIPPING) FUSION	• RAS/RAF/MEK/ MAPK • PI3K/AKT/MTOR	• MET inhibitors	e.g. **crizotinib, capmatinib	NCT03297606; NCT02219711
**CDK6 MUTATION**	• Cell cycle pathway	• CDK4/CDK6 inhibitors	e.g. ** palbociclib, ribociclib	NCT01037790; NCT03297606
IDH1 MUTATION *TIER 1A DIAGNOSTIC* *TIER 1A PROGNOSTIC*	• DNA Repair • Gene expression regulation	• IDH1 inhibitors • PARP inhibitors • Immunotherapy	*ivosidenib e.g. **olaparib, rucaparib, talazoparib e.g. **pembrolizumab, nivolumab	NCT03557359; NCT03207347
**MYC AMPLIFICATION**	• Transcriptional regulation of gene expression	• PI3K inhibitors • AKT Inhibitors • MTOR inhibitors • CHEK 1/2 / ATR inhibitors	e.g. **alpelisib, capivasertib, everolimus	NCT02873975; NCT03718091
**RB1 MUTATION**	• Cell cycle pathway	• CDK4/CDK6 inhibitors	e.g. ** palbociclib, ribociclib	NCT02873975; NCT01037790
**RB1 DELETION**	• Cell cycle pathway	• CDK4/CDK6 inhibitors	e.g. ** palbociclib, ribociclib	NCT02873975; NCT01037790
**ARID1A DELETION**	• Gene expression regulation/DNA Repair	• PARP inhibitors	e.g. **olaparib, rucaparib, talazoparib	NCT02286687
**CDKN2B DELETION**	• Cell cycle pathway	• CDK4/CDK6 inhibitors	e.g. ** palbociclib, ribociclib	NCT01037790
**CREBBP MUTATION**	• Gene expression regulation	• CBP/p300 inhibitors	e.g. **CSS1477	NCT03568656
**TP53 MUTATION**	• Cell cycle/DNA repair	• Cell cycle checkpoint inhibitors • PARP inhibitors	e.g. ** e.g. **olaparib, rucaparib, talazoparib	NCT02576444
**CDK4 AMPLIFICATION**	• Cell cycle pathway	• CDK4/CDK6 inhibitors	e.g** abemaciclib, everolimus, ribociclib, palbociclib	NCT03220646; NCT03834740; NCT03310879; NCT02693535; NCT03994796; NCT03297606; NCT01037790
**ATR DELETION**	• DNA repair	• PARP inhibitor • PARP inhibitor + immunotherapy	e.g***durvalumab + Olaparib, Olaparib, Talazoparib	NCT02286687; NCT03233204
**NBN DELETION**	• DNA repair	• PARP inhibitor • PARP inhibitor + immunotherapy	e.g** talazoparib, Olaparib, pamiparib, tislelizumab	NCT02286687; NCT03233204; NCT02660034
**CDK6 AMPLIFICATION**	• Cell cycle pathway	• CDK4/CDK6 inhibitors	e.g** everolimus, ribociclib, abemaciclib, palbociclib,	NCT03834740; NCT03310879; NCT02693535; NCT03994796; NCT01037790
**MDM2 AMPLIFICATION**	• Gene expression regulation	• MDM2 inhibitors	e.g** ATSP-7041, BI 907828	NCT03654716; NCT03449381
**PIK3CB MUTATION**	• PI3K/AKT/MTOR pathway	• PI3K inhibitors • AKT Inhibitors • MTOR inhibitors	e.g ** paxalisib, samotolisib, atezolizumab + ipatasertib, AZD-8186, chemotherapy, gedatolisib + palbociclib	NCT03994796; NCT03155620; NCT03213678; NCT03673787; NCT03218826; NCT03065062
**TMEM178B-BRAF FUSION**	• RAS/RAF/MEK/ERK	• RAS inhibitors • RAF inhibitors • MEK inhibitors • MAPK inhibitors	e.g** cobimetinib, trametinib, ulixertinib, binimetinib, encorafenib, ASTX029, mirdametinib, lifirafenib, LXH254, LY3214996, midazolam, abemaciclib, chemotherapy, encorafenib, cetuximab, RMC-463	NCT02639546; NCT03363217; NCT02465060; NCT03843775; NCT03520075; NCT03905148; NCT02607813; NCT02857270; NCT03634982
**TBL1XR1—PIK3CA FUSION**	• PI3K/AKT/MTOR pathway	• PI3K inhibitors • AKT Inhibitors • MTOR inhibitors	e.g** temsirolimus, capivasertib, olaparib, atezolizumab + ipatasertib, gedatolisib + palbociclib	NCT03297606; NCT02576444; NCT03673787; NCT03065062
**CAPZA2 –MET FUSION**	• RAS/RAF/MEK/ERK • PI3K/AKT	• MET inhibitors	e.g**volitinib, crizotinib, bozitinib, sitravatinib	NCT03598244; NCT03297606; NCT03175224; NCT02219711
**PTPRZ1 –MET FUSION**	• RAS/RAF/MEK/ERK • PI3K/AKT	• MET inhibitors	e.g** volitinib, crizotinib, bozitinib, sitravatinib	NCT03598244; NCT03297606; NCT03175224; NCT02219711

Analysis of actionable variants detected in our cohort were cross referenced with captured genetic alterations in previous cohort studies to determine whether the actionable mutational landscape observed in this study is in keeping with previous genomic findings in glioblastoma. Potentially actionable variants identified in 46 glioblastoma samples using the JAX ActionSeq™ NGS Panel correlated significantly with our findings [21]. The overall frequency of actionable SNVs identified in this study were tightly correlated with their frequency in the JAX study (r2 = 0.88), TP53, PTEN, IDH1 and NF1 variants being identified at high frequency in both studies. Data collected by exome or whole genome sequencing from The Cancer Genome Atlas showed less correlation (r2 = 0.58) [22]. In particular, IDH1 variants were identified at a much lower frequency (5.2%) than in our study (14.5%), while EGFR variants were found at a higher frequency (21.0% vs 9.1%).

Increased levels of PD-L1 tumour cell expression were also identified in a significant proportion of glioblastoma cases. Using a defined predictive cut point of >10% for tumour proportion score, identified as a cut-point for PD-L1 IHC companion diagnostic assays in relation to anti-PD-L1/anti PD-1 directed therapies [23], PD-L1 expression levels were significantly raised in 27.3% of glioblastomas with 12.7% of cases having a tumour proportion score of >50% (S4 Fig). Moreover, actionable variants were identified in 43.6% of patients with aberrations in the DNA repair genes BRCA2 (3.6%), ATM (3.6%), NBN (3.6%), ATR (3.6%), MLH1 (1.8%), ATRX (7.3%), SETD2 (3.6%), PTEN (21.8%), and IDH1 (14.5%). These DNA repair genes share in common evidence-based linkage to anti-PD-L1/PD-1 immunotherapies including pembrolizumab, nivolumab, atezolizumab, avelumab, durvalumab and tislelizumab and PARP inhibitors (Table 1). The total percentage of glioblastoma patients with either DNA repair defects or elevated PD-L1 levels amounted to 63.6% of cases and 7.3% of cases were associated with both a DNA repair defect and elevated PD-L1 levels.

## Discussion

Advances in our understanding of somatic cancer genetics and immuno-oncology are now being rapidly translated into clinical practice particularly for advanced metastatic solid tumours. There is now a shift in treatment paradigm from the relatively non-specific empirically directed cytotoxic chemotherapies to a more biologically informed approach where oncogenic somatic mutations are matched with the appropriate targeted agents [24–26]. The drug-target pairing that links a dysregulated molecular pathway with a cognate therapeutic agent defines the modern era of precision oncology. This approach has demonstrated superior response rates as compared to nonselective chemotherapy in many tumour types and is associated with less toxicity [12, 13].

Analysis of actionable genomic profiling trending data has revealed unique insights into the complex actional mutational landscape of glioblastoma, the underlying pathobiology that drives this aggressive tumour phenotype and the wide range of potential targeted treatment options available. Actionable variants were identified in all glioblastoma patients tested and indeed the majority of patients harboured three or more actionable variants. This compares to our analysis of solid tumours overall where actionable variants were detected in 90% of patients with a median variant frequency of two (median 2, range 0–13) [27]. The genetic variants identified affect many key cancer-related regulatory networks including the PI3K/AKT/MTOR, RAS/RAF/MEK/MAPK, JAK/STAT, PLC/PKC signalling pathways, DNA damage repair (DDR) pathways and cell cycle and immune checkpoints. These are key signalling networks mediating fundamental processes including cell proliferation, differentiation, cell migration, DNA damage response and apoptosis which are involved in homeostasis across all tissue types [28–30]. This may account for the fact that the majority of detected actionable genomic variants are independent of tumour type or tissue of origin. The high frequency and the presence of multiple actionable mutations identified for each patient tested highlights the broad range of potential personalized treatment options that are available for the treatment of this aggressive disease (Table 1). Many of the targeted agents are directed against the aberrant oncoprotein directly, for example erdafitinib against mutated FGFR1 and FGFR3 and imatinib and sunitinib targeting KIT amplification. Alternative treatment protocols have also been developed targeting components of the cognate signalling pathways downstream. For example, the TBL1XR1-PIK3CA fusion is targeted with therapies inhibiting PI3K directly (alpelisib) or alternatively using inhibitors downstream including AKT (capivasertib), MTOR (temsirolimus) and CDK4/6 (abemaciclib) (Table 1).

Although actionable fusion genes are a relatively rare occurrence in most tumour types [20, 31], here we show that glioblastoma harbours a high frequency of actionable oncogenic fusion genes, in 23.6% of cases. This is in keeping with the observation that mRNA fusions transcripts, including non-druggable fusions, have been identified at high frequency (65%) in glioblastoma [32] Notably all fusions share in common interaction with one or more of the major oncogenic signalling pathways namely RAS/RAF/MEK/MAPK, PI3K/AKT/MTOR, JAK/STAT or PLC/PKC signalling cascades (Table 1). This includes the two novel fusions genes we have identified in glioblastoma, TBL1XR1-PIK3CA and FIP1L1-PDGFRA. Interestingly, the mean number of actionable mutations was greater in samples containing fusions or deletions. This may relate to the fact that fusions and deletions are an indicator of a high degree of cancer cell genome instability [20, 33]. Moreover, fusion tyrosine kinases have been shown to compromise the fidelity of DNA repair mechanisms which can lead to the accumulation of additional genetic aberrations thus functioning as “first hit” oncogenic aberrations [34–36]. A potential linkage was also observed between genomic instability as indicated by high TMB, increased frequency of actionable mutations and loss of DDR function. This is in keeping with observations in other tumour types such as non-small cell lung cancer showing that aberrations in DDR genes strongly correlate with high TMB [37]. Here we have also shown that glioblastomas defective in DDR function have a higher frequency of p53 mutations and that IDH1 mutant tumours are associated with a higher frequency of actionable variants.

Concurrent mutations were observed for a number of the actionable genes identified in our cohort. The simultaneous deletions of CDKN2A and CDKN2B is frequently identified across many tumour types as a consequence of homozygous 9p21.3 deletions involving the CDKN2A/p14ARF/CDKN2B loci [38]. Concurrent alterations of EGFR A289V and EGFR Amplification, IDH1 R132H and PIK3R1 N564D and IDH1 R132H and TP53 R273C have been described previously as signatures of mutation and selection in the cancer genome [39]. Concurrent mutations in IDH1 and ATRX have been described previously in glioblastoma and appear more prevalent in tumours without receptor tyrosine kinase (RTK) activation [32].

A wide range of DNA repair genes were also found to be mutated in this cohort of glioblastoma patients including ATR, ATM, ATRX, IDH1, BRCA2, NBN, PTEN, SETD2 and the MMR related gene MLH1. These aberrations were bioinformatically linked to either monotherapy with PARP inhibitors harnessing a synthetic lethality approach to treatment or alternatively anti-PD-L1/PD-1/CTLA-4 directed immunotherapy or in combination (Table 1). The linkage with immunotherapy is a consequence that deleterious mutations in DNA repair genes can give rise to an increase in neoantigens [40]. Neoantigens in turn are able to elicit tumour-specific immune responses which can then be amplified by the immune-activating actions of immunotherapy including inhibitors of CTLA-4, PD-1 and PD-L1. This linkage is highlighted in the recent landmark FDA approval for PD-1 inhibitors in MMR-deficient tumours [41]. Combination therapy with PARP inhibitors and immunotherapy is a compelling strategy and early trials utilizing this strategy are showing encouraging results [42].

Around 8–23% of glioblastoma patients show response to anti-PD-L1/PD-1 directed immunotherapies and the challenge now is to identify more effective companion diagnostic markers that can further refine identification of this treatment responsive group [43]. In keeping with previous studies we observed varying levels of upregulation of PD-L1 in a high proportion of (62%) of cases [44]. PD-L1 expression levels have been identified as a predictor of response in some tumour types but is a relatively poor biomarker of response due to the fact it is not a static biomarker and does not offer binary discrimination of responsiveness [45, 46]. Interestingly a subpopulation of cases in our cohort (7%) showed both elevated PD-L1 expression levels and aberration of DNA repair genes and we are currently investigating whether this signature can be used as a potential improved predictor of immune response across all tumour types [47].

Notwithstanding the high frequency of actionable variants observed in our cohort, EGFR amplification was detected at the lower end frequency to that previously reported. The frequency of reported EGFR amplification is highly variable (8–50%) and is influenced by many parameters including ethnicity and glioblastoma subtype [32, 48, 49]. The relatively lower frequency observed in our cohort may be a consequence of the fact that samples were sourced globally from different ethnic populations and also included secondary glioblastomas in which EGFR amplification events are a rare event [48, 49].

In summary we have shown that comprehensive semiconductor precision oncology profiling for actionable variants can significantly increase the potential therapeutic armamentarium available for the treatment of glioblastoma compared to the biomarkers currently used in routine clinical practice and notably targeted therapies in clinical trials are showing promising results [50–56]. Interestingly, the majority of actionable genomic variants identified were not tumour type specific, reinforcing the “site agnostic” approach to genomic profiling and supporting the concept of “molecular basket” clinical trials [57]. The adoption of semiconductor sequencing methodologies enables clinically directed targeted precision oncology profiling to be applied robustly to routine FFPE clinical biopsy samples allowing integration with established routine diagnostic pathology workflows.

## Supporting information

S1 Table Patient demographics. (PDF)

S2 Table Performance characteristics of the oncofocus test. (PDF)

S3 Table List of genes tested. (PDF)

S4 Table Quality control metrics. (PDF)

S5 Table List of SNVs detected by the assay. (PDF)

S6 Table List of CNVs detected by the assay. (PDF)

S7 Table List of gene fusions detected by the assay. (PDF)

S8 Table Evidence base for targeted therapies crossing the blood brain barrier. (PDF)

S1 Fig Landscape of actionable mutations. (TIF)

S2 Fig Stacked bar graph showing frequency of samples that have between 0–9 actionable alterations. (TIF)

S3 Fig Frequency of variant type in glioblastoma. (TIF)

S4 Fig PD-L1 expression in glioblastoma. (TIF)
