# Supplementary material for: Utilisation of semiconductor sequencing for the detection of predictive biomarkers in glioblastoma
Source: PLoS One. 2022 Mar 24;17(3):e0245817. doi: 10.1371/journal.pone.0245817 (PMC8947072; doi:10.1371/journal.pone.0245817)
Supplement: S1 Table — (PDF) [file pone.0245817.s001.pdf]

Supplementary Table 1. Patient demographics.

	N= (% of samples)
**Sex:**	
• **Males**	37 (67.3)
• **Females**	18 (32.7)
**Age (years):**	
• **Mean**	49.7
• **Median**	50
• **Range**	60 (min. 11, max. 71)
**(Standard deviation)**	15.2
**Recurrence:**	
• **Primary**	44 (80)
• **Secondary**	3 (5.5)
• **Recurrent**	11 (20)
**IDH1 (Codon 132) Status**	
**Tested in 52 samples**	
• **Wild-type**	44 (84.6)
• **Mutant**	8 (15.4)
**IDH2 (Codon 172) Status**	
**Tested in 12 samples**	
• **Wild-type**	12 (100)
• **Mutant**	0 (0)
**MGMT Promoter Methylation:**	
**Tested in 47 samples**	
• **Methylated**	19 (40.4)
• **Unmethylated**	28 (59.6)
**1p/19q status: Tested in 18 samples**	
**1p:**	
• **Retained**	18 (100)
• **Lost**	0 (0)
**19q:**	
• **Retained**	16 (88.9)
• **Lost**	0 (0)
