# Supplementary material for: Utilisation of semiconductor sequencing for the detection of predictive biomarkers in glioblastoma
Source: PLoS One. 2022 Mar 24;17(3):e0245817. doi: 10.1371/journal.pone.0245817 (PMC8947072; doi:10.1371/journal.pone.0245817)
Supplement: S2 Table — (PDF) [file pone.0245817.s002.pdf]

Supplementary Table 2. Performance characteristics of the Oncofocus test.

	SNVs	Indel	CNV	Fusions
**PPV***	>99%	>99%	>99%	>99%
**NPV****	>99%	>99%	>99%	>99%
**Accuracy**	>99%	>99%	>99%	>99%
**Sensitivity**	>99%	89%	>99%	>99%
**Specificity**	>99%	>99%	>99%	>99%

*Positive predictive value

**Negative predictive value
