# Supplementary material for: Utilisation of semiconductor sequencing for the detection of predictive biomarkers in glioblastoma
Source: PLoS One. 2022 Mar 24;17(3):e0245817. doi: 10.1371/journal.pone.0245817 (PMC8947072; doi:10.1371/journal.pone.0245817)
Supplement: S4 Table — (PDF) [file pone.0245817.s004.pdf]

Supplementary Table 4. Quality control metrics.

Quality Metric	Controlled parameters	Sample
Chip loading	≥60%	95%
Total reads	>45 million reads	100,480,836
Enrichment	100% ideal	100%
Clonal: Polyclonal ratio	Up to 55% polyclonal	25%
Low Quality	≤26% approximately.	7%
Usable reads	≥30%	70%
Aligned bases	≥80% Can be less if base coverage, and % reads on target is high	87%
Unaligned bases	≤20%	13%
Mean raw accuracy	This value should be as close to 100% as possible	99%
Overall read length histogram	Median read length 98-115bp for DNA only, 65-90bp RNA only, DNA & RNA 100bp ±20	113

DNA

Quality Metric	Controlled parameters	Sample
Number of mapped reads	>4.5 million	10,580,325
Percent reads on target	>90%	94.65%
Average base coverage depth	>1200	2,981
Uniformity of amplicon (base) coverage	>90% (degraded 85-90%)	94.60%
Percent assigned amplicon reads	>90%	94.65%
Amplicons with no strand bias	>90%	97.38%
Amplicons reading end to end	>80%	94.79%
% base reads on target	>85%	91.04%
coverage at 1X	>90% coverage is required at 500X for samples with tumour percentages below 40%	99.96%
coverage at 20X		99.68%
coverage at 100X		98.78%
coverage at 500X		95.43%
MAPD value	In the presence of a copy number variant, this value should be <0.5	0.305

RNA

Quality Metric	Controlled parameters	Test specific figures
Mapped reads	>40,000 for software to call a fusion, (should be over 500,000)	1,430,677
Expression Control Genes MYC	>15 read counts indicates the gene is present (5 out of 6 genes should be present to accept the presence of a fusion)	74670
Expression Control Genes HMBS		1706
Expression Control Genes TBP		11083
Expression Control Genes LRP1		2487
Expression Control Genes ITGB7		3732
Expression Control Genes MRPL13		1331
