# Supplementary material for: Utilisation of semiconductor sequencing for the detection of predictive biomarkers in glioblastoma
Source: PLoS One. 2022 Mar 24;17(3):e0245817. doi: 10.1371/journal.pone.0245817 (PMC8947072; doi:10.1371/journal.pone.0245817)
Supplement: S5 Table — (PDF) [file pone.0245817.s005.pdf]

Supplementary Table 5. List of SNVs detected by the assay.

ONC No	Cancer Type	Locus	Genotype	Ref	Observed Allele	Type	Gene	Transcript	Variant Class	Gene Class	Amino Acid Change	Coverage	% Allele Frequency	Exon	Coding	Variant Effect	PhyloP	SIFT	Grantham	PolyPhen	PFAM	dbSNP
ONC18 0141	Glioblastoma	chr2:209113112	CG/TG	CG	AG,TG,AC,GA	SNV	IDH1	NM_005896.3	Hotspot	Gain-of-function	p.Arg132His	1995	39.75	4	c.395G>A	missense	1.36,2.62	0	29	0.127	Isocitrate/isopropylmalate dehydrogenase	rs121913499,rs121913500
ONC18 0141	Glioblastoma	chr9:98220308	G/A	G	A	SNV	PTCH1	NM_000264.3	Hotspot	Loss-of-function	p.Thr1052Met	1987	49.22	18	c.3155C>T	missense	2.94	0.04	81	0.951	Patched family	rs138911275
ONC18 0288	Glioblastoma	chr10:89653807	G/A	G	A	SNV	PTEN	NM_000314.6	Hotspot	Loss-of-function	p.Met35Ile	1397	17.97	2	c.105G>A	missense	9.02		10	0.969	Dual specificity phosphatase, catalytic domain	rs786202517
ONC18 0292	Glioblastoma	chr7:55221822	C/T	C	A,T	SNV	EGFR	NM_005228.3	Hotspot	Gain-of-function	p.Ala289Val	1998	85.54	7	c.866C>T	missense	7.8	0	64	0.998	Furin-like cysteine rich region,Protein kinase domain,Protein tyrosine kinase,Receptor L domain	rs149840192
ONC18 0292	Glioblastoma	chr17:7579358	C/A	C	A	SNV	TP53	NM_000546.5	Hotspot	Loss-of-function	p.Arg110Leu	946	27.59	4	c.329G>T	missense	0.56	0.05	102	0.373	P53 DNA-binding domain,P53 tetramerisation motif	rs11540654,rs587780066,rs587782490
ONC18 0338	Glioblastoma	chr17:29661945	C/T	C	T	SNV	NF1	NM_001042492.2	Deleterious	Loss-of-function	p.Arg1968Ter	1915	35.3	40	c.5902C>T	nonsense	2.87				GTPase-activator protein for Ras-like GTPase	rs137854552
ONC18 0338	Glioblastoma	chr17:7577574	T/C	T	A,C,G	SNV	TP53	NM_000546.5	Hotspot	Loss-of-function	p.Tyr236Cys	983	55.44	7	c.707A>G	missense	1.5	0	194	0.999	P53 DNA-binding domain,P53 tetramerisation motif	rs730882026,rs794727846
ONC18 0339	Glioblastoma	chr17:7577505	T/A	T	A	SNV	TP53	NM_000546.5	Hotspot	Loss-of-function	p.Asp259Val	1407	41.22	7	c.776A>T	missense	2.62	0.04	152	0.537	P53 DNA-binding domain,P53 tetramerisation motif	rs745425759
ONC18 0631	Glioblastoma	chr2:209113111	AC/AT	AC	AT,GT	SNV	IDH1	NM_005896.3	Hotspot	Gain-of-function	p.Arg132His	767	43.16	4	c.395G>A	missense	0.25, 6.09	0	29	0.127	Isocitrate/isopropylmalate dehydrogenase	rs121913500,rs753058442
ONC18 0631	Glioblastoma	chr5:67591097	A/G	A	G	SNV	PIK3R1	NM_181523.2	Hotspot	Loss-of-function	p.Asn564Asp	2000	4.4	13	c.1690A>G	missense	9.09	0	23	0.933	Phosphatidylinositol 3-kinase regulatory subunit P85 inter-SH2 domain,RhoGAP domain,SH2 domain	rs1057519840,rs1057519841,rs758509723
ONC18 0631	Glioblastoma	chr17:7577121	G/A	G	A,C,T	SNV	TP53	NM_000546.5	Hotspot	Loss-of-function	p.Arg273Cys	1192	33.47	8	c.817C>T	missense	4.54	0	180	1	P53 DNA-binding domain,P53 tetramerisation motif	rs121913343,rs587781987
ONC18 0638	Glioblastoma	chr7:55221822	C/T	C	A,T	SNV	EGFR	NM_005228.4	Hotspot	Gain-of-function	p.Ala289Val	1999	13.11	7	c.866C>T	missense	7.8	0	64	0.998	Furin-like cysteine rich region,Growth factor receptor domain IV,Protein kinase domain,Protein tyrosine kinase,Receptor L domain	rs1057519887,rs149840192
ONC18 0661	Glioblastoma	chr17:7578403	C/A	C	A,G,T	SNV	TP53	NM_000546.5	Hotspot	Loss-of-function	p.Cys176Phe	2000	69.75	5	c.527G>T	missense	7.78	0	205	1	P53 DNA-binding domain,P53 tetramerisation motif	rs786202962
ONC18 0739	Glioblastoma	chr2:209113113	G/T	G	A,C,T	SNV	IDH1	NM_005896.3	Hotspot	Gain-of-function	p.Arg132Ser	974	38.3	4	c.394C>A	missense	7.06	0	110	0.981	Isocitrate/isopropylmalate dehydrogenase	rs121913499,rs753058442
ONC18 0739	Glioblastoma	chr17:7577121	G/A	G	A,C,T	SNV	TP53	NM_000546.5	Hotspot	Loss-of-function	p.Arg273Cys	1865	81.02	8	c.817C>T	missense	4.54	0	180	1	P53 DNA-binding domain,P53 tetramerisation motif	rs121913343,rs587781987
ONC18 0743	Glioblastoma	chr11:108128243	GT/G	GT	G	INDEL	ATM	NM_000051.3	Deleterious	Loss-of-function	p.Phe763fs	1985	50.03	15	c.2289delT	frameshiftDeletion	0.31, 3.67				Anaphase-promoting complex subunit 5,FAT domain,FATC domain,Phosphatidylinositol 3- and 4-kinase,Telomere-length maintenance and DNA damage repair	rs1064795831
ONC18 0743	Glioblastoma	chr3:178952085	A/G	A	C,G,T	SNV	PIK3CA	NM_006218.3	Hotspot	Gain-of-function	p.His1047Arg	977	37.15	21	c.3140A>G	missense	8.86	0.03	29	0.639	PI3-kinase family, p85-binding domain,PI3-kinase family, ras-binding domain,Phosphatidylinositol 3- and 4-kinase,Phosphoinositide 3-kinase C2,Phosphoinositide 3-kinase family, accessory domain (PIK domain)	rs121913279
ONC18 0792	Glioblastoma	chr13:48941637	ATCTT/A	ATCTT	A	INDEL	RB1	NM_000321.2	Deleterious	Loss-of-function	p.Ser318fs	981	12.54	10	c.951_954delTTCT	frameshiftDeletion	0.27, 2.39, 1.59, 5.81, -0.47				Domain of unknown function (DUF3452),Rb C-terminal domain,Retinoblastoma-associated protein A domain,Retinoblastoma-associated protein B domain	rs749023632
ONC18 0793	Glioblastoma	chr2:209113111	AC/AT	AC	AT,GT	SNV	IDH1	NM_005896.3	Hotspot	Gain-of-function	p.Arg132His	1288	22.59	4	c.395G>A	missense	0.25, 6.09	0	29	0.127	Isocitrate/isopropylmalate dehydrogenase	rs121913500,rs753058442
ONC18 0793	Glioblastoma	chr17:7577121	G/A	G	A,C,T	SNV	TP53	NM_000546.5	Hotspot	Loss-of-function	p.Arg273Cys	1999	42.42	8	c.817C>T	missense	4.54	0	180	1	P53 DNA-binding domain,P53 tetramerisation motif	rs121913343,rs587781987
ONC18 0793	Glioblastoma	chrX:76940000	G/A	G	A	SNV	ATRX	NM_000489.4	Deleterious	Loss-of-function	p.Arg250Ter	673	20.51	9	c.748C>T	nonsense	1.31				Helicase conserved C-terminal domain,SNF2 family N-terminal domain	
ONC18 0827	Glioblastoma	chr7:55211080	G/A	G	A	SNV	EGFR	NM_005228.4	Hotspot	Gain-of-function	p.Arg108Lys	2000	92.4	3	c.323G>A	missense	9.79	0	26	1	Furin-like cysteine rich region,Growth factor receptor domain IV,Protein kinase domain,Protein tyrosine kinase,Receptor L domain	rs1057519828
ONC18 0929	Glioblastoma	chr2:209113111	AC/AT	AC	AT,GT	SNV	IDH1	NM_005896.3	Hotspot	Gain-of-function	p.Arg132His	931	34.37	4	c.395G>A	missense	0.25, 6.09	0	29	0.127	Isocitrate/isopropylmalate dehydrogenase	rs121913500,rs753058442
ONC18 0929	Glioblastoma	chr17:7577580	T/C	T	A,C,G	SNV	TP53	NM_000546.5	Hotspot	Loss-of-function	p.Tyr234Cys	1001	25.07	7	c.701A>G	missense	2.04	0	194	0.999	P53 DNA-binding domain,P53 tetramerisation motif	rs587780073
ONC18 0931	Glioblastoma	chr5:67589138	G/A	G	A,C	SNV	PIK3R1	NM_181523.2	Hotspot	Loss-of-function	p.Gly376Arg	678	32.89	10	c.1126G>A	missense	9.61	0	125	1	Phosphatidylinositol 3-kinase regulatory subunit P85 inter-SH2 domain,RhoGAP domain,SH2 domain	rs1057519757
ONC18 0933	Glioblastoma	chr17:7574003	G/A	G	A	SNV	TP53	NM_000546.5	Deleterious	Loss-of-function	p.Arg342Ter	1742	22.79	10	c.1024C>T	nonsense	2.17				P53 DNA-binding domain,P53 tetramerisation motif	rs730882029
ONC18 0933	Glioblastoma	chr5:67591116	T/C	T	C	SNV	PIK3R1	NM_181523.2	Hotspot		p.Leu570Pro	1304	21.01	13	c.1709T>C	missense	7.83	0	98	1	Phosphatidylinositol 3-kinase regulatory subunit P85 inter-SH2 domain,RhoGAP domain,SH2 domain	
ONC18 0963	Glioblastoma	chr5:67589138	G/A	PASS	G	SNV	PIK3R1	NM_181523.2	Hotspot	Loss-of-function	p.Gly376Arg	1108	31.05	10	c.1126G>A	missense	9.61	0	125	1	Phosphatidylinositol 3-kinase regulatory subunit P85 inter-SH2 domain,RhoGAP domain,SH2 domain	rs1057519757
ONC18 1020	Glioblastoma	chr2:209113111	AC/AT	AC	AT,GT	SNV	IDH1	NM_005896.3	Hotspot	Gain-of-function	p.Arg132His	1172	43.86	4	c.395G>A	missense	0.25, 6.09	0	29	0.127	Isocitrate/isopropylmalate dehydrogenase	rs121913500,rs753058442
ONC18 1020	Glioblastoma	chr12:112888199	C/T	C	A,T	SNV	PTPN11	NM_002834.4	Hotspot	Gain-of-function	p.Ala72Val	1305	9.96	3	c.215C>T	missense	7.81	0	64	0.987	Protein-tyrosine phosphatase,SH2 domain	rs121918454
ONC18 1020	Glioblastoma	chr17:7577121	G/A	G	A,C,T	SNV	TP53	NM_000546.5	Hotspot	Loss-of-function	p.Arg273Cys	1633	85.24	8	c.817C>T	missense	4.54	0	180	1	P53 DNA-binding domain,P53 tetramerisation motif	rs121913343,rs587781987
ONC18 1020	Glioblastoma	chrX:76938390	AGATGTAGA/A	AGATGTAGA	A	INDEL	ATRX	NM_000489.4	Deleterious	Loss-of-function	p.Ser784fs	1157	83.58	9	c.2350_2357delTCTACATC	frameshiftDeletion	0.19, 8.72, 6.54, 2.81, 1.52, 1.75, 1.99, 3.32, 5.55				Helicase conserved C-terminal domain,SNF2 family N-terminal domain	rs782333412
ONC18 1053	Glioblastoma	chr17:7577538	C/T	C	A,G,T	SNV	TP53	NM_000546.5	Hotspot	Loss-of-function	p.Arg248Gln	2000	86.2	7	c.743G>A	missense	5.89	0.01	43	1	P53 DNA-binding domain,P53 tetramerisation motif	rs11540652
ONC18 1053	Glioblastoma	chr3:47165329	TG/T	TG	T,TT	INDEL	SETD2	NM_014159.6	Deleterious	Loss-of-function	p.His266fs	1984	87.7	3	c.796delC	frameshiftDeletion	0.86, 4.72				SET domain,SRI (Set2 Rpb1 interacting) domain,WW domain	
ONC19 0012	Glioblastoma	chr17:7577568	C/T	C	A,G,T	SNV	TP53	NM_000546.5	Hotspot	Loss-of-function	p.Cys238Tyr	1801	88.62	7	c.713G>A	missense	7.56	0	194	1	P53 DNA-binding domain,P53 tetramerisation motif	rs730882005
ONC19 0012	Glioblastoma	chr10:89653785	T/C	T	C	SNV	PTEN	NM_000314.6	Hotspot	Loss-of-function	p.Ile28Thr	1046	75.14	2	c.83T>C	missense	7.34	0	89	0.999	C2 domain of PTEN tumour-suppressor protein,Dual specificity phosphatase, catalytic domain	
ONC19 0016	Glioblastoma	chr2:209113111	AC/AT	AC	AT,GT	SNV	IDH1	NM_005896.3	Hotspot	Gain-of-function	p.Arg132His	1996	40.83	4	c.395G>A	missense	0.25, 6.09	0	29	0.127	Isocitrate/isopropylmalate dehydrogenase	rs121913500,rs753058442
ONC19 0016	Glioblastoma	chr3:37061817	C/T	C	T	SNV	MLH1	NM_000249.3	Deleterious	Loss-of-function	p.Gln301Ter	1732	28.12	11	c.901C>T	nonsense	3.76				DNA mismatch repair protein Mlh1 C-terminus,DNA mismatch repair protein, C-terminal domain,Histidine kinase-, DNA gyrase B-, and HSP90-like ATPase	rs63750489
ONC19 0016	Glioblastoma	chr17:29508779	C/T	C	T	SNV	NF1	NM_001042492.2	Deleterious	Loss-of-function	p.Gln236Ter	1031	40.06	7	c.706C>T	nonsense	4.2				Divergent CRAL/TRIO domain,GTPase-activator protein for Ras-like GTPase	
ONC19 0016	Glioblastoma	chr5:67591097	A/G	A	G	SNV	PIK3R1	NM_181523.2	Hotspot	Loss-of-function	p.Asn564Asp	2000	18.15	13	c.1690A>G	missense	9.09	0	23	0.933	Phosphatidylinositol 3-kinase regulatory subunit P85 inter-SH2 domain,RhoGAP domain,SH2 domain	rs1057519840,rs1057519841,rs758509723
ONC19 0016	Glioblastoma	chr10:89717727	G/A	G	A	SNV	PTEN	NM_000314.6	Hotspot	Loss-of-function	p.Gly251Asp	2000	47.25	7	c.752G>A	missense	9.43	0	94	0.999	C2 domain of PTEN tumour-suppressor protein,Dual specificity phosphatase, catalytic domain	
ONC19 0016	Glioblastoma	chr17:7578208	T/A	T	A,C,G	SNV	TP53	NM_000546.5	Hotspot	Loss-of-function	p.His214Leu	1996	41.93	6	c.641A>T	missense	8	0	99	0.997	P53 DNA-binding domain,P53 tetramerisation motif	rs1057519992
ONC19 0016	Glioblastoma	chr17:7577121	G/A	G	A,C,T	SNV	TP53	NM_000546.5	Hotspot	Loss-of-function	p.Arg273Cys	2000	40.7	8	c.817C>T	missense	4.54	0	180	1	P53 DNA-binding domain,P53 tetramerisation motif	rs121913343,rs587781987
ONC19 0018	Glioblastoma	chr13:32972626	A/T	A	T	SNV	BRCA2	NM_000059.3	Deleterious	Loss-of-function	p.Lys3326Ter	1176	44.98	27	c.9976A>T	nonsense	0.52				BRCA2 repeat,BRCA2, helical,BRCA2, oligonucleotide/oligosaccharide-binding, domain 1,BRCA2, oligonucleotide/oligosaccharide-binding, domain 3,Tower	rs11571833
ONC19 0036	Glioblastoma	chr10:89692904	C/T	C	G,T	SNV	PTEN	NM_000314.6	Deleterious	Loss-of-function	p.Arg130Ter	1997	77.62	5	c.388C>T	nonsense	5.53				C2 domain of PTEN tumour-suppressor protein,Dual specificity phosphatase, catalytic domain	rs121909224,rs121913292
ONC19 0036	Glioblastoma	chr17:7578442	T/C	T	C,G	SNV	TP53	NM_000546.5	Hotspot	Loss-of-function	p.Tyr163Cys	1999	55.63	5	c.488A>G	missense	5.14	0	194	1	P53 DNA-binding domain,P53 tetramerisation motif	rs148924904
ONC19 0061	Glioblastoma	chr3:138374298	A/C	A	C	SNV	PIK3CB	NM_006219.2	Hotspot	Gain-of-function	p.Leu1049Arg	1333	17.03	22	c.3146T>G	missense	9.14	0	102	0.989	PI3-kinase family, p85-binding domain,PI3-kinase family, ras-binding domain,Phosphatidylinositol 3- and 4-kinase,Phosphoinositide 3-kinase C2,Phosphoinositide 3-kinase family, accessory domain (PIK domain)	
ONC19 0061	Glioblastoma	chr7:140453133	TTCA/TTCT	TTCA	ATCA,CTCA,GTCA,T,TTCT	SNV	BRAF	NM_004333.4	Hotspot	Gain-of-function	p.Val600Glu	855	14.5	15	c.1799T>A	missense	7.92, 7.92, 4.01, 9.2	0	121	0.971	Kinase-like,Phorbol esters/diacylglycerol binding domain (C1 domain),Protein kinase domain,Protein tyrosine kinase,Raf-like Ras-binding domain	rs113488022,rs121913226,rs121913227,rs121913364,rs121913377,rs397507484,rs397516897
ONC19 0066	Glioblastoma	chr7:55221822	C/T	C	A,T	SNV	EGFR	NM_005228.4	Hotspot	Gain-of-function	p.Ala289Val	1999	3.65	7	c.866C>T	missense	7.8	0	64	0.998	Furin-like cysteine rich region,Growth factor receptor domain IV,Protein kinase domain,Protein tyrosine kinase,Receptor L domain	rs1057519887,rs149840192
ONC19 0066	Glioblastoma	chr4:153268144	G/A	G	A	SNV	FBXW7	NM_033632.3	Deleterious	Loss-of-function	p.Arg222Ter	1999	33.97	4	c.664C>T	nonsense	4.63				F-box domain,F-box-like,PQQ-like domain,WD domain, G-beta repeat	rs920052554
ONC19 0066	Glioblastoma	chr1:27105725	A/G	A	G	SNV	ARID1A	NM_006015.5	Hotspot	Loss-of-function	p.Glu1779Gly	1998	46.25	20	c.5336A>G	missense	3.64		98	0.483	ARID/BRIGHT DNA binding domain,SWI/SNF-like complex subunit BAF250/Osa	rs140946580
ONC19 0088	Glioblastoma	chr9:135778003	G/A	G	A	SNV	TSC1	NM_000368.4	Deleterious	Loss-of-function	p.Gln794Ter	1997	31.95	18	c.2380C>T	nonsense	9.32				Hamartin protein	rs781371665
ONC19 0088	Glioblastoma	chr11:108138003	T/C	T	C	SNV	ATM	NM_000051.3	Hotspot	Loss-of-function	p.Phe858Leu	1996	42.54	17	c.2572T>C	missense	2.92		22	0.732	Anaphase-promoting complex subunit 5,FAT domain,FATC domain,Phosphatidylinositol 3- and 4-kinase,Telomere-length maintenance and DNA damage repair	rs1800056
ONC19 0088	Glioblastoma	chr17:7579415	C/T	C	T	SNV	TP53	NM_000546.5	Deleterious	Loss-of-function	p.Trp91Ter	1975	44.25	4	c.272G>A	nonsense	3.72				P53 DNA-binding domain,P53 tetramerisation motif	rs886041861
ONC19 0137	Glioblastoma	chr10:89692985	G/T	G	T	SNV	PTEN	NM_000314.6	Deleterious	Loss-of-function	p.Glu157Ter	705	54.18	5	c.469G>T	nonsense	9.43				C2 domain of PTEN tumour-suppressor protein,Dual specificity phosphatase, catalytic domain	rs121909220
ONC19 0137	Glioblastoma	chr13:48954373	T/G	T	G	SNV	RB1	NM_000321.2	Deleterious	Loss-of-function	p.Tyr498Ter	1461	45.38	16	c.1494T>G	nonsense	2.88				Domain of unknown function (DUF3452),Rb C-terminal domain,Retinoblastoma-associated protein A domain,Retinoblastoma-associated protein B domain	rs768929728
ONC19 0137	Glioblastoma	chr17:7578395	G/A	G	A,C,T	SNV	TP53	NM_000546.5	Hotspot	Loss-of-function	p.His179Tyr	1998	51.65	5	c.535C>T	missense	9.82	0	83	0.999	P53 DNA-binding domain,P53 tetramerisation motif	rs587780070
ONC19 0186	Glioblastoma	chr10:89717695	CT/C	CT	C	INDEL	PTEN	NM_000314.6	Deleterious	Loss-of-function	p.Phe241fs	1519	55.43	7	c.723delT	frameshiftDeletion	1.48, 7.66				C2 domain of PTEN tumour-suppressor protein,Dual specificity phosphatase, catalytic domain	rs1060500115,rs190070312
ONC19 0202	Glioblastoma	chr4:1806131	T/C	T	C	SNV	FGFR3	NM_000142.4	Hotspot	Gain-of-function	p.Phe384Leu	620	50.16	9	c.1150T>C	missense	0.28	0.44	22	0.003	Immunoglobulin I-set domain,Immunoglobulin domain,Protein kinase domain,Protein tyrosine kinase	rs17881656
ONC19 0202	Glioblastoma	chr7:55221822	C/T	C	A,T	SNV	EGFR	NM_005228.4	Hotspot	Gain-of-function	p.Ala289Val	1995	11.83	7	c.866C>T	missense	7.8	0	64	0.998	Furin-like cysteine rich region,Growth factor receptor domain IV,Protein kinase domain,Protein tyrosine kinase,Receptor L domain	rs1057519887,rs149840192
ONC19 0225	Glioblastoma	chr3:178916876	G/A	G	A	SNV	PIK3CA	NM_006218.3	Hotspot	Gain-of-function	p.Arg88Gln	1998	16.92	2	c.263G>A	missense	7.57	0	43	1	PI3-kinase family, p85-binding domain,PI3-kinase family, ras-binding domain,Phosphatidylinositol 3- and 4-kinase,Phosphoinositide 3-kinase C2,Phosphoinositide 3-kinase family, accessory domain (PIK domain)	rs121913287
ONC19 0225	Glioblastoma	chr17:29422358	C/T	C	T	SNV	NF1	NM_001042492.2	Deleterious	Loss-of-function	p.Gln11Ter	1670	15.51	1	c.31C>T	nonsense	2.85				Divergent CRAL/TRIO domain,GTPase-activator protein for Ras-like GTPase	rs876658658
ONC19 0234	Glioblastoma	chr17:7577538	T/T	C	A,G,T	SNV	TP53	NM_000546.5	Hotspot	Loss-of-function	p.Arg248Gln	2000	97.3	5.89	c.743G>A	missense	5.89	0.01	43	1	P53 DNA-binding domain,P53 tetramerisation motif	rs11540652
ONC19 0234	Glioblastoma	chr16:3808019	CCATGGGATTCTTTA/C	CCATGGGATTCTTTA	C	INDEL	CREBBP	NM_004380.2	Deleterious	Loss-of-function	p.Val1129fs	1913	42.81	18	c.3386_3399delTAA	frameshiftDeletion	7.72, 2.53, 9.21, 7.93, 0.16, 9.73, 9.73, 1.33, 4.02, 7.93, 2.66, 7.93, 7.93, 3.22, 9.21				Bromodomain,Creb binding,Domain of Unknown Function (DUF902),Histone acetylation protein,KIX domain,TAZ zinc finger,Zinc finger, ZZ type	rs749030378,rs770547195
ONC19 0234	Glioblastoma	chrX:76888721	ATTTC/A	ATTTC	A	INDEL	ATRX	NM_000489.4	Deleterious	Loss-of-function	p.Glu1702fs	1975	52.66	19	c.5104_5107delGAAA	frameshiftDeletion	5.9, 4.77, 2.47, 7.67, 7.46				Helicase conserved C-terminal domain,SNF2 family N-terminal domain	
ONC19 0272	Glioblastoma	chr17:29562746	C/T	C	T	SNV	NF1	NM_001042492.2	Deleterious	Loss-of-function	p.Arg1276Ter	1974	77.76	28	c.3826C>T	nonsense	3.22				Divergent CRAL/TRIO domain,GTPase-activator protein for Ras-like GTPase	rs199474742
ONC19 0371	Glioblastoma	chr1:115256529	TG/TT	TG	AA,AG,CG,CT,GG,TT	SNV	NRAS	NM_002524.4	Hotspot	Gain-of-function	p.Gln61Lys	1996	15.28	3	c.181C>A	missense	7.76, 9.52	0.01	53	0.948	ADP-ribosylation factor family,Ras family,Ras of Complex, Roc, domain of DAPkinase	rs1057519695,rs1057519834,rs11554290,rs121913254
ONC19 0418	Glioblastoma	chr2:209113111	AC/AT	AC	AT,GT	SNV	IDH1	NM_005896.3	Hotspot	Gain-of-function	p.Arg132His	1861	37.08	4	c.395G>A	missense	0.25, 6.09	0	29	0.127	Isocitrate/isopropylmalate dehydrogenase	rs121913500,rs753058442
ONC19 0418	Glioblastoma	chr17:7578524	G/C	G	C	SNV	TP53	NM_000546.5	Hotspot	Loss-of-function	p.Gln136Glu	1999	74.89	5	c.406C>G	missense	9.81	0	29	0.996	P53 DNA-binding domain,P53 tetramerisation motif	rs786203589
ONC19 0418	Glioblastoma	chrX:76939700	TG/T	TG	T	INDEL	ATRX	NM_000489.4	Deleterious	Loss-of-function	p.Glu351fs	1981	75.57	9	c.1047delC	frameshiftDeletion	7.7, 0.26				Helicase conserved C-terminal domain,SNF2 family N-terminal domain	rs200288042
ONC19 0428	Glioblastoma	chr13:49033906	G/A	G	A	SNV	RB1	NM_000321.2	Deleterious	Loss-of-function	p.Trp681Ter	1526	19.66	20	c.2043G>A	nonsense	9.48				Domain of unknown function (DUF3452),Rb C-terminal domain,Retinoblastoma-associated protein A domain,Retinoblastoma-associated protein B domain	
ONC19 0428	Glioblastoma	chr17:29665751	CACTT/C	CACTT	C	INDEL	NF1	NM_001042492.2	Deleterious	Loss-of-function	p.Tyr2285fs	1515	28.71	46	c.6852_6855delTTAC	frameshiftDeletion	1.01, 6.84, 5.09, 2.4, 7.42				Divergent CRAL/TRIO domain,GTPase-activator protein for Ras-like GTPase	rs747881072,rs863224836,rs876657715
ONC19 0437	Glioblastoma	chr10:89692849	G/A	G	A	SNV	PTEN	NM_000314.6	Deleterious	Loss-of-function	p.Trp111Ter	1999	7.5	5	c.333G>A	nonsense	9.43				C2 domain of PTEN tumour-suppressor protein,Dual specificity phosphatase, catalytic domain	
ONC19 0437	Glioblastoma	chr17:29528086	C/G	C	G	SNV	NF1	NM_001042492.2	Deleterious	Loss-of-function	p.Ser365Ter	939	7.88	10	c.1094C>G	nonsense	3.64				Divergent CRAL/TRIO domain,GTPase-activator protein for Ras-like GTPase	rs864622107
ONC19 0468	Glioblastoma	chr17:29486075	GA/G	GA	G	INDEL	NF1	NM_001042492.2	Deleterious	Loss-of-function	p.Ile85fs	1755	47.92	3	c.253delA	frameshiftDeletion	3.35, 8.63				Divergent CRAL/TRIO domain,GTPase-activator protein for Ras-like GTPase	
ONC19 0472	Glioblastoma	chr13:49027222	C/T	C	T	SNV	RB1	NM_000321.2	Deleterious	Loss-of-function	p.Gln597Ter	1900	63.79	18	c.1789C>T	nonsense	6.15				Domain of unknown function (DUF3452),Rb C-terminal domain,Retinoblastoma-associated protein A domain,Retinoblastoma-associated protein B domain	
ONC19 0472	Glioblastoma	chr17:7577094	G/A	G	A,C	SNV	TP53	NM_000546.5	Hotspot	Loss-of-function	p.Arg282Trp	2000	78.35	8	c.844C>T	missense	4.48	0	101	1	P53 DNA-binding domain,P53 tetramerisation motif	rs28934574,rs587781564
ONC19 0532	Glioblastoma	chr10:89720789	G/T	G	T	SNV	PTEN	NM_000314.6	Deleterious	Loss-of-function	p.Glu314Ter	902	87.8	8	c.940G>T	nonsense	9.19				C2 domain of PTEN tumour-suppressor protein,Dual specificity phosphatase, catalytic domain	rs764753843
ONC19 0599	Glioblastoma	chr12:112888198	G/A	G	A,T	SNV	PTPN11	NM_002834.4	Hotspot	Gain-of-function	p.Ala72Thr	1999	A=43.92, T=0.00	3	c.214G>A	missense	9.87	0	58	0.996	Protein-tyrosine phosphatase,SH2 domain	rs121918453
ONC19 0599	Glioblastoma	chr8:38274849	G/T	G	C,T	SNV	FGFR1	NM_001174067.1	Hotspot	Gain-of-function	p.Asn577Lys	1998	44.79	13	c.1731C>A	missense	2	0	94	1	Immunoglobulin I-set domain,Immunoglobulin domain,Protein kinase domain,Protein tyrosine kinase	rs779707422
ONC19 0599	Glioblastoma	chr3:178952085	A/G	A	C,G,T	SNV	PIK3CA	NM_006218.3	Hotspot	Gain-of-function	p.His1047Arg	1998	45.95	21	c.3140A>G	missense	8.86	0.03	29	0.639	PI3-kinase family, p85-binding domain,PI3-kinase family, ras-binding domain,Phosphatidylinositol 3- and 4-kinase,Phosphoinositide 3-kinase C2,Phosphoinositide 3-kinase family, accessory domain (PIK domain)	rs121913279
ONC19 0611	Glioblastoma	chr3:47143030	G/A	G	A	SNV	SETD2	NM_014159.6	Deleterious	Loss-of-function	p.Gln1645Ter	2000	21.05	8	c.4933C>T	nonsense	9.77				SET domain,SRI (Set2 Rpb1 interacting) domain,WW domain	
ONC19 0611	Glioblastoma	chr17:29664894	C/T	C	T	SNV	NF1	NM_001042492.2	Deleterious	Loss-of-function	p.Gln2234Ter	1072	17.44	44	c.6700C>T	nonsense	7.22				Divergent CRAL/TRIO domain,GTPase-activator protein for Ras-like GTPase	
ONC19 0611	Glioblastoma	chr12:112926909	A/T	A	T	SNV	PTPN11	NM_002834.4	Hotspot	Gain-of-function	p.Gln510Leu	1513	9.85	13	c.1529A>T	missense	8.91	0	113	1	Protein-tyrosine phosphatase,SH2 domain	rs121918470
ONC19 0611	Glioblastoma	chr10:89692883	C/T	C	G,T	SNV	PTEN	NM_000314.6	Hotspot	Loss-of-function	p.His123Tyr	1999	40.17	5	c.367C>T	missense	7.45	0	83	0.999	C2 domain of PTEN tumour-suppressor protein,Dual specificity phosphatase, catalytic domain	rs786204931
ONC19 0611	Glioblastoma	chr17:7577575	A/T	A	C,G,T	SNV	TP53	NM_000546.5	Hotspot	Loss-of-function	p.Tyr236Asn	1999	38.62	7	c.706T>A	missense	9.08	0	143	1	P53 DNA-binding domain,P53 tetramerisation motif	rs587782289,rs794727846
