# Supplementary material for: Utilisation of semiconductor sequencing for the detection of predictive biomarkers in glioblastoma
Source: PLoS One. 2022 Mar 24;17(3):e0245817. doi: 10.1371/journal.pone.0245817 (PMC8947072; doi:10.1371/journal.pone.0245817)
Supplement: S6 Table — (PDF) [file pone.0245817.s006.pdf]

Supplementary Table 6. List of CNVs detected by the assay.

ONC number	Locus	Type	Genes	Length	Variant Class	Gene Class	Copy Number	CytoBand	CNV Confidence			
ONC18 0125	chr7:55211009	CNV	EGFR	57.082kb	Amplification	Gain-of-function	87.89	7p11.2(55211009-55268091)x87.8857	5%	72.9	95%	105.92
ONC18 0135	chr7:55211009	CNV	EGFR	57.082kb	Amplification	Gain-of-function	23.46	7p11.2(55211009-55268091)x23.4556	5%	20.04	95%	27.45
ONC18 0141	chr8:128748723	CNV	MYC	4.475kb	Amplification	Gain-of-function	6.86	8q24.21(128748723-128753198)x6.86316	5%	5.59	95%	8.42
ONC18 0146	chr9:21968185	CNV	CDKN2A	26.204kb	Deletion	Loss of function	0.02	9p21.3(21968185-21994389)x0.0235294	5%	0	95%	0.08
ONC18 0146	chr4:55589727	CNV	KIT	13.721kb	Amplification	Gain-of-function	28.84	4q12(55589727-55603448)x28.8353	5%	25.58	95%	32.5
ONC18 0146	chr4:55141006	CNV	PDGFRA	14.29kb	Amplification	Gain-of-function	39.84	4q12(55141006-55155296)x39.8353	5%	34.83	95%	45.55
ONC18 0292	chr7:55211009	CNV	EGFR	57.082kb	Amplification	Gain-of-function	36.09	7p11.2(55211009-55268091)x36.0875	5%	31.88	95%	40.84
ONC18 0338	chr9:135771565	CNV	TSC1	32.735kb	Deletion	Loss of function	0.82	9q34.13(135771565-135804300)x0.816667	5%	0.67	95%	0.97
ONC18 0339	chr7:55211009	CNV	EGFR	57.082kb	Amplification	Gain-of-function	66.38	7p11.2(55211009-55268091)x66.375	5%	59.02	95%	74.64
ONC18 0401	chr12:58142244	CNV	CDK4	3.206kb	Amplification	Gain-of-function	87.74	12q14.1(58142244-58145450)x87.7429	5%	73.45	95%	104.79
ONC18 0638	chr4:55141007	CNV	PDGFRA	14.289kb	Amplification	Gain-of-function	6.41	4q12(55141007-55155296)x6.41053	5%	5.58	95%	7.36
ONC18 0638	chr7:55211010	CNV	EGFR	57.081kb	Amplification	Gain-of-function	38.37	7p11.2(55211010-55268091)x38.3684	5%	33.75	95%	43.62
ONC18 0661	chr9:21968186	CNV	CDKN2A	26.203kb	Deletion	Loss of function	0.3	9p21.3(21968186-21994389)x0.3	5%	0.23	95%	0.38
ONC18 0739	chr13:32890491	CNV	BRCA2	82.441kb	Deletion	Loss of function	0.8	13q13.1(32890491-32972932)x0.8	5%	0.74	95%	0.86
ONC18 0743	chr9:21968186	CNV	CDKN2A	26.203kb	Deletion	Loss of function	0.78	9p21.3(21968186-21994389)x0.778947	5%	0.68	95%	0.89
ONC18 0793	chr3:142168234	CNV	ATR	129.327kb	Deletion	Loss of function	0.43	3q23(142168234-142297561)x0.433333	5%	0.17	95%	0.71
ONC18 0793	chr8:90947804	CNV	NBN	49.011kb	Deletion	Loss of function	0.3	8q21.3(90947804-90996815)x0.3	5%	0	95%	0.79
ONC18 0827	chr9:21968186	CNV	CDKN2A	26.203kb	Deletion	Loss of function	0.05	9p21.3(21968186-21994389)x0.0526316	5%	0.03	95%	0.08
ONC18 0827	chr9:22005844	CNV	CDKN2B	3.135kb	Deletion	Loss of function	0.02	9p21.3(22005844-22008979)x0.0210526	5%	0	95%	0.04
ONC18 0827	chr7:55211010	CNV	EGFR	57.081kb	Amplification	Gain-of-function	49.97	7p11.2(55211010-55268091)x49.9684	5%	44.07	95%	56.65
ONC18 0833	chr9:21968186	CNV	CDKN2A	26.203kb	Deletion	Loss of function	0.51	9p21.3(21968186-21994389)x0.506667	5%	0.34	95%	0.7
ONC18 0833	chr9:22005844	CNV	CDKN2B	3.135kb	Deletion	Loss of function	0.47	9p21.3(22005844-22008979)x0.466667	5%	0.3	95%	0.67
ONC18 0833	chr10:89624208	CNV	PTEN	100.953kb	Deletion	Loss of function	0.59	10q23.31(89624208-89725161)x0.586667	5%	0.43	95%	0.76
ONC18 0931	chr1:27022880	CNV	ARID1A	84.399kb	Deletion	Loss of function	0.79	1p36.11(27022880-27107279)x0.785714	5%	0.67	95%	0.91
ONC18 0948	chr7:55211010	CNV	EGFR	57.081kb	Amplification	Gain-of-function	44.03	7p11.2(55211010-55268091)x44.0333	5%	39.2	95%	49.44
ONC18 0948	chr9:21968186	CNV	CDKN2A	26.203kb	Deletion	Loss of function	0.22	9p21.3(21968186-21994389)x0.216667	5%	0.01	95%	0.45
ONC18 0948	chr9:22005844	CNV	CDKN2B	3.135kb	Deletion	Loss of function	0.12	9p21.3(22005844-22008979)x0.116667	5%	0	95%	0.35
ONC18 0963	chr7:55211010	CNV	EGFR	57.081kb	Amplification	Gain-of-function	50.86	7p11.2(55211010-55268091)x50.8556	5%	42.71	95%	60.55
ONC18 1053	chr7:92244372	CNV	CDK6	218.246kb	Amplification	Gain-of-function	27.69	7q21.2(92244372-92462618)x27.6947	5%	23.67	95%	32.4
ONC18 1053	chr7:116339592	CNV	MET	96.459kb	Amplification	Gain-of-function	18.25	7q31.2(116339592-116436051)x18.2526	5%	16.37	95%	20.35
ONC19 0016	chr9:21968186	CNV	CDKN2A	26.203kb	Deletion	Loss of function	0.24	9p21.3(21968186-21994389)x0.242105	5%	0.19	95%	0.3
ONC19 0016	chr9:22005844	CNV	CDKN2B	3.135kb	Deletion	Loss of function	0.21	9p21.3(22005844-22008979)x0.210526	5%	0.16	95%	0.28
ONC19 0036	chr4:55141007	CNV	PDGFRA	14.289kb	Amplification	Gain-of-function	17.19	4q12(55141007-55155296)x17.1882	5%	15.15	95%	19.49
ONC19 0036	chr12:58142245	CNV	CDK4	3.205kb	Amplification	Gain-of-function	28.95	12q14.1(58142245-58145450)x28.9529	5%	24.84	95%	33.74
ONC19 0066	chr7:55211010	CNV	EGFR	57.081kb	Amplification	Gain-of-function	33.54	7p11.2(55211010-55268091)x33.5429	5%	28.52	95%	39.42
ONC19 0066	chr9:22005844	CNV	CDKN2B	3.135kb	Deletion	Loss of function	0.07	9p21.3(22005844-22008979)x0.0714286	5%	0	95%	0.28
ONC19 0179	chr7:55211010	CNV	EGFR	57.081kb	Amplification	Gain-of-function	11.77	7p11.2(55211010-55268091)x11.7714	5%	9.78	95%	14.13
ONC19 0179	chr9:21968186	CNV	CDKN2A	26.203kb	Deletion	Loss of function	0.13	9p21.3(21968186-21994389)x0.128571	5%	0	95%	0.34
ONC19 0179	chr9:22005844	CNV	CDKN2B	3.135kb	Deletion	Loss of function	0.21	9p21.3(22005844-22008979)x0.214286	5%	0	95%	0.48
ONC19 0186	chr7:55211010	CNV	EGFR	57.081kb	Amplification	Gain-of-function	30.35	7p11.2(55211010-55268091)x30.3474	5%	26.47	95%	34.79
ONC19 0186	chr9:22005844	CNV	CDKN2B	3.135kb	Deletion	Loss of function	0.34	9p21.3(22005844-22008979)x0.336842	5%	0.26	95%	0.42
ONC19 0202	chr7:55211010	CNV	EGFR	57.081kb	Amplification	Gain-of-function	26.38	7p11.2(55211010-55268091)x26.3789	5%	22.33	95%	31.16
ONC19 0202	chr9:21968186	CNV	CDKN2A	26.203kb	Deletion	Loss of function	0.34	9p21.3(21968186-21994389)x0.336842	5%	0.26	95%	0.43
ONC19 0234	chr3:142168234	CNV	ATR	129.327kb	Deletion	Loss of function	0.88	3q23(142168234-142297561)x0.877778	5%	0.83	95%	0.92
ONC19 0234	chr8:90947804	CNV	NBN	49.011kb	Deletion	Loss of function	0.63	8q21.3(90947804-90996815)x0.633333	5%	0.57	95%	0.7
ONC19 0234	chr9:21968186	CNV	CDKN2A	26.203kb	Deletion	Loss of function	0	9p21.3(21968186-21994389)x0	5%	0	95%	0
ONC19 0234	chr9:22005844	CNV	CDKN2B	3.135kb	Deletion	Loss of function	0	9p21.3(22005844-22008979)x0	5%	0	95%	0
ONC19 0244	chr7:55211010	CNV	EGFR	57.081kb	Amplification	Gain-of-function	77.92	7p11.2(55211010-55268091)x77.9222	5%	65.76	95%	92.33
ONC19 0244	chr9:21968186	CNV	CDKN2A	26.203kb	Deletion	Loss of function	0.14	9p21.3(21968186-21994389)x0.144444	5%	0.08	95%	0.22
ONC19 0284	chr9:21968186	CNV	CDKN2A	26.203kb	Deletion	Loss of function	0.2	9p21.3(21968186-21994389)x0.2	5%	0.13	95%	0.29
ONC19 0284	chr10:89624208	CNV	PTEN	100.953kb	Deletion	Loss of function	0.21	10q23.31(89624208-89725161)x0.211111	5%	0.14	95%	0.29
ONC19 0284	chr13:48877966	CNV	RB1	176.264kb	Deletion	Loss of function	0.62	13q14.2(48877966-49054230)x0.622222	5%	0.54	95%	0.71
ONC19 0335	chr12:58142245	CNV	CDK4	3.205kb	Amplification	Gain-of-function	8.07	12q14.1(58142245-58145450)x8.075	5%	6.35	95%	10.12
ONC19 0335	chr12:69202191	CNV	MDM2	31.261kb	Amplification	Gain-of-function	7.75	12q15(69202191-69233452)x7.75	5%	5.99	95%	9.85
ONC19 0351	chr7:55211010	CNV	EGFR	57.081kb	Amplification	Gain-of-function	31.11	7p11.2(55211010-55268091)x31.1143	5%	27.48	95%	35.22
ONC19 0351	chr10:89624208	CNV	PTEN	100.953kb	Deletion	Loss of function	0	10q23.31(89624208-89725161)x0	5%	0	95%	0
ONC19 0441	chr12:58142245	CNV	CDK4	3.205kb	Amplification	Gain-of-function	5.61	12q14.1(58142245-58145450)x5.6125	5%	4.48	95%	7.01
ONC19 0468	chr9:21968186	CNV	CDKN2A	26.203kb	Deletion	Loss of function	0.41	9p21.3(21968186-21994389)x0.413333	5%	0.25	95%	0.61
ONC19 0472	chr4:55141007	CNV	PDGFRA	14.289kb	Amplification	Gain-of-function	8.19	4q12(55141007-55155296)x8.1875	5%	6.92	95%	9.67
ONC19 0472	chr4:55589728	CNV	KIT	13.72kb	Amplification	Gain-of-function	6.19	4q12(55589728-55603448)x6.1875	5%	5.31	95%	7.19
ONC19 0472	chr4:55955798	CNV	KDR	17.148kb	Deletion	Loss of function	7.54	4q12(55955798-55972946)x7.5375	5%	5.43	95%	10.4
ONC19 0473	chr12:58142245	CNV	CDK4	3.205kb	Amplification	Gain-of-function	5.91	12q14.1(58142245-58145450)x5.9125	5%	4.82	95%	7.22
ONC19 0532	chr9:21968186	CNV	CDKN2A	26.203kb	Deletion	Loss of function	0.21	9p21.3(21968186-21994389)x0.210526	5%	0.17	95%	0.26
