# Supplementary material for: Utilisation of semiconductor sequencing for the detection of predictive biomarkers in glioblastoma
Source: PLoS One. 2022 Mar 24;17(3):e0245817. doi: 10.1371/journal.pone.0245817 (PMC8947072; doi:10.1371/journal.pone.0245817)
Supplement: S7 Table — (PDF) [file pone.0245817.s007.pdf]

Supplementary Table 7. List of gene fusions detected by the assay.

ONC number	Locus	Ref	Type	Genes	Variant Class	Variant ID	Read Counts	Exon (gene 1)	Exon (gene 2)
ONC18 0146	chr4:54294802 - chr4:55141107	C	FUSION	FIP1L1(13) - PDGFRA(12)	Fusion	FIP1L1-PDGFRA.F13ins11P12del99	101	13	12
ONC18 0146	chr7:116538889 - chr7:116339125	G	FUSION	CAPZA2(4) - MET(2)	Fusion	CAPZA2-MET.C4M2.Non-Targeted	8004	4	2
ONC18 0339	chr7:55087058 - chr7:55223523	G	RNAExonVariant	EGFR(1) - EGFR(8)	Fusion	EGFR-EGFR.E1E8.DelPositive.2	26217	1	8
ONC18 0401	chr4:1808661 - chr4:1732899	C	FUSION	FGFR3(17) - TACC3(6)	Fusion	FGFR3-TACC3.F17T6	115731	17	6
ONC18 0638	chr7:55268106 - chr7:55863785	G	FUSION	EGFR(24) - SEPT14(10)	Fusion	EGFR-SEPT14.E24S10	25476	24	10
ONC18 1053	chr7:116502704 - chr7:116395409	G	FUSION	CAPZA2(1) - MET(6)	Fusion	CAPZA2-MET.C1M6	97	1	6
ONC18 1053	chr7:116411708 - chr7:116414935	G	RNAExonVariant	MET(13) - MET(15)	RNAExonVariant	MET-MET.M13M15	1450	13	15
ONC19 0066	chr7:55087058 - chr7:55223523	G	RNAExonVariant	EGFR(1) - EGFR(8)	RNAExonVariant	EGFR-EGFR.E1E8.DelPositive.2	84070	1	8
ONC19 0186	chr7:55268106 - chr7:55863785	G	FUSION	EGFR(24) - SEPT14(10)	Fusion	EGFR-SEPT14.E24S10	228049	24	10
ONC19 0202	chr7:55268106 - chr7:55863785	G	FUSION	EGFR(24) - SEPT14(10)	Fusion	EGFR-SEPT14.E24S10	157146	24	10
ONC19 0244	chr7:121513611 - chr7:116339125	G	FUSION	PTPRZ1(1) - MET(2)	Fusion	PTPRZ1-MET.P1M2	312	1	2
ONC19 0244	chr7:55087058 - chr7:55223523	G	RNAExonVariant	EGFR(1) - EGFR(8)	RNAExonVariant	EGFR-EGFR.E1E8.DelPositive.2	1372	1	8
ONC19 0335	chr4:1808661 - chr4:1732899	C	FUSION	FGFR3(17) - TACC3(6)	Fusion	FGFR3-TACC3.F17T6	279210	17	6
ONC19 0418	chr3:176914909 - chr3:178916538	C	FUSION	TBL1XR1(1) - PIK3CA(2)	Fusion	TBL1XR1-PIK3CA.T1P2	77	1	2
ONC19 0418	chr7:121513611 - chr7:116339125	G	FUSION	PTPRZ1(1) - MET(2)	Fusion	PTPRZ1-MET.P1M2	36161	1	2
ONC19 0437	chr7:141255367 - chr7:140494267	G	FUSION	AGK(2) - BRAF(8)	Fusion	AGK-BRAF.A2B8	310	2	8
ONC19 0534	chr7:140912504 - chr7:140487384		FUSION	TMEM178B(2) - BRAF(9)	Fusion	TMEM178B-BRAF.T2B9	1662	2	9
