# Supplementary material for: Utilisation of semiconductor sequencing for the detection of predictive biomarkers in glioblastoma
Source: PLoS One. 2022 Mar 24;17(3):e0245817. doi: 10.1371/journal.pone.0245817 (PMC8947072; doi:10.1371/journal.pone.0245817)
Supplement: S8 Table — (PDF) [file pone.0245817.s008.pdf]

RMC-4630	No data	
rucaparib	No	Breast Cancer Resistance Protein (BCRP/ABCG2) and P-glycoprotein (P-GP/ABCB1) Restrict Oral Availability and Brain Accumulation of the PARP Inhibitor Rucaparib (AG-014699) | SpringerLink
samotolisib	Potentially - awaiting trial data	Samotolisib in Treating Patients With Relapsed or Refractory Advanced Solid Tumors, Non-Hodgkin Lymphoma, or Histiocytic Disorders With TSC or PI3K/MTOR Mutations (A Pediatric MATCH Treatment Trial) - Full Text View - ClinicalTrials.gov
Savolitinib	Yes	Development and characterization of patient-derived xenografts from non-small cell lung cancer brain metastases | Scientific Reports (nature.com)
sitravatinib	No data	
sorafenib	Yes	Molecularly targeted therapies for recurrent glioblastoma: current and future targets in: Neurosurgical Focus Volume 37 Issue 6 (2014) (thejns.org)
sunitinib	Yes	Molecularly targeted therapies for recurrent glioblastoma: current and future targets in: Neurosurgical Focus Volume 37 Issue 6 (2014) (thejns.org)
talazoparib	No	MCT-17-0365 2735..2746 (aacrjournals.org)
TAS-120 (futibatinib)	Potentially - awaiting trial data	A Study of TAS-120 in Patients With Advanced Solid Tumors - Full Text View - ClinicalTrials.gov
temsirolimus	Limited	Molecularly targeted therapies for recurrent glioblastoma: current and future targets in: Neurosurgical Focus Volume 37 Issue 6 (2014) (thejns.org)
tislelizumab	Potentially - based on binding of FcRn receptor	
trametinib	No	Brain Distribution of a Novel MEK Inhibitor E6201: Implications in the Treatment of Melanoma Brain Metastases (nih.gov)
ulixertinib	Potentially - awaiting trial data	Ulixertinib in Treating Patients With Advanced Solid Tumors, Non-Hodgkin Lymphoma, or Histiocytic Disorders With MAPK Pathway Mutations (A Pediatric MATCH Treatment Trial) - Full Text View - ClinicalTrials.gov
vemurafenib	No	Brain Distribution of a Novel MEK Inhibitor E6201: Implications in the Treatment of Melanoma Brain Metastases (nih.gov)
vismodegib	Yes	Vismodegib Exerts Targeted Efficacy Against Recurrent Sonic Hedgehog–Subgroup Medulloblastoma: Results From Phase II Pediatric Brain Tumor Consortium Studies PBTC-025B and PBTC-032 (nih.gov)

Immunotherapy Drugs with FcRn receptors and BBB	https://www.ncbi.nlm.nih.gov/pmc/articles/PMC6543612/<br>https://journals.sagepub.com/doi/full/10.1177/1756286421997381<br>https://www.mdpi.com/1422-0067/22/12/6442

Small molecules and BB	Drug transport across the blood–brain barrier (nih.gov)

**yes**	**29**
**limited**	**12**
**no**	**6**
**unclear/no data**	**11**
**Drugs**	**58**
