# Supplementary figures and images for: Utilisation of semiconductor sequencing for the detection of predictive biomarkers in glioblastoma
Source: PLoS One. 2022 Mar 24;17(3):e0245817. doi: 10.1371/journal.pone.0245817 (PMC8947072; doi:10.1371/journal.pone.0245817)

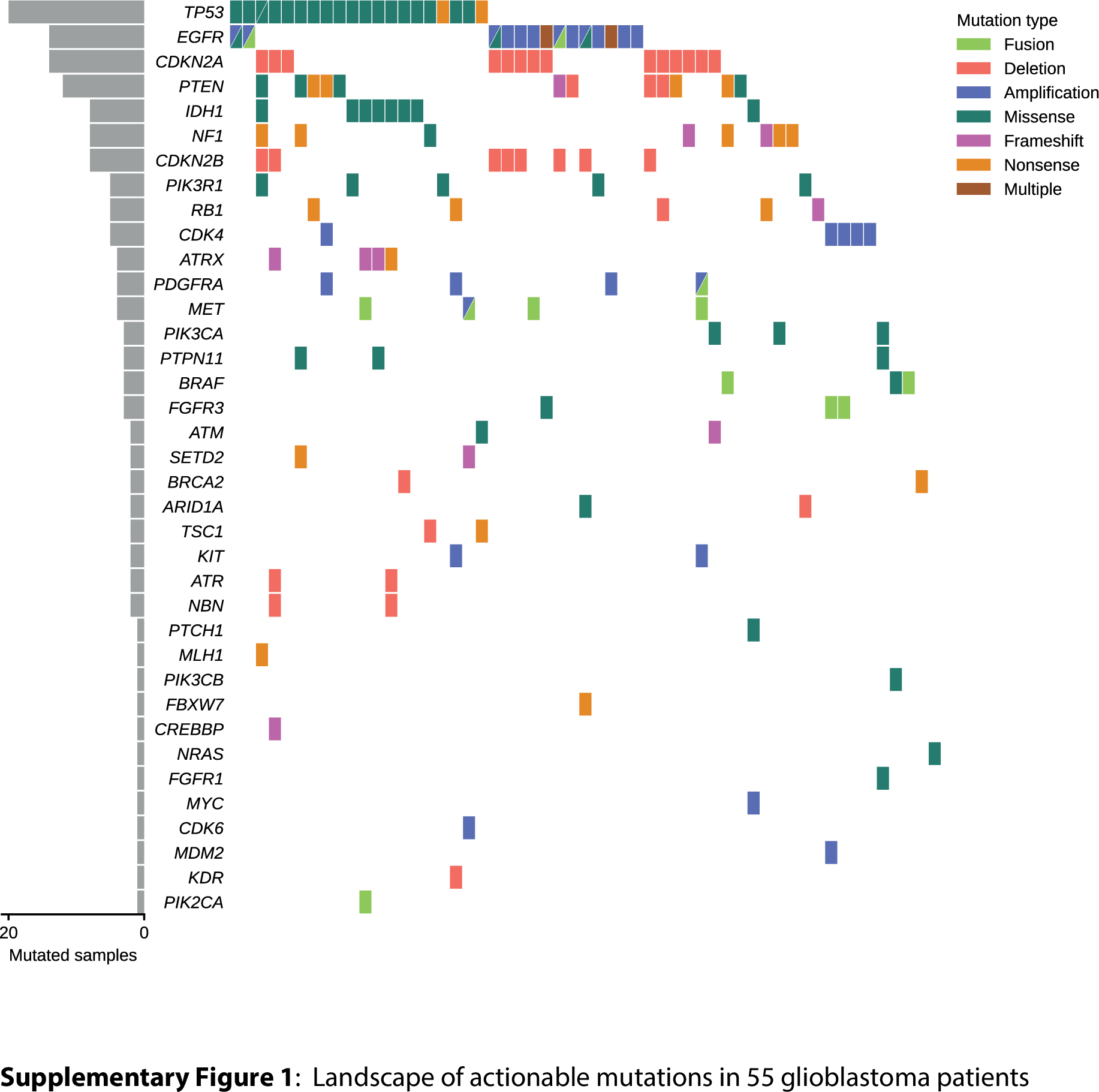

Supplement: S1 Fig — (TIF) [file pone.0245817.s009.tif]

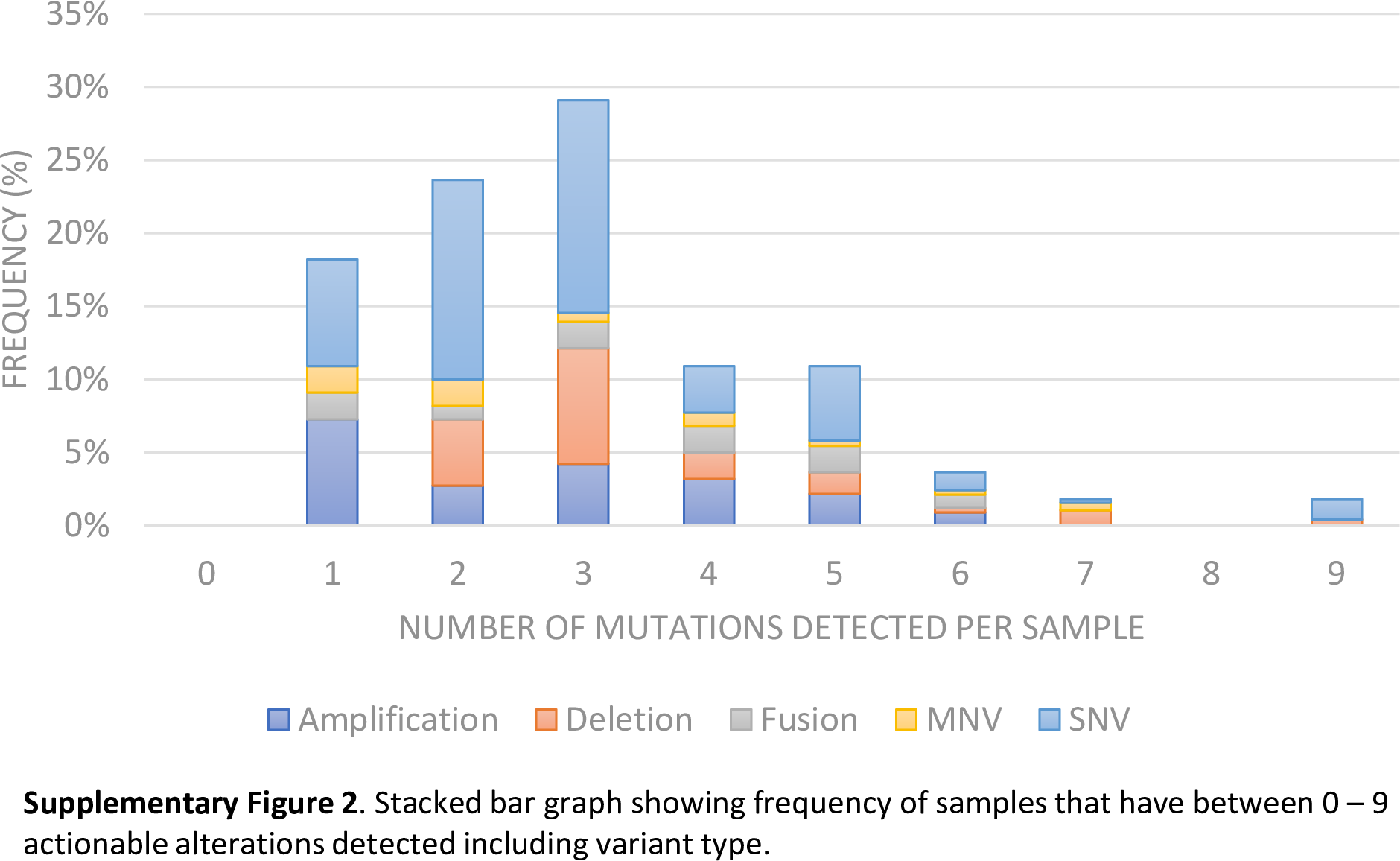

Supplement: S2 Fig — (TIF) [file pone.0245817.s010.tif]

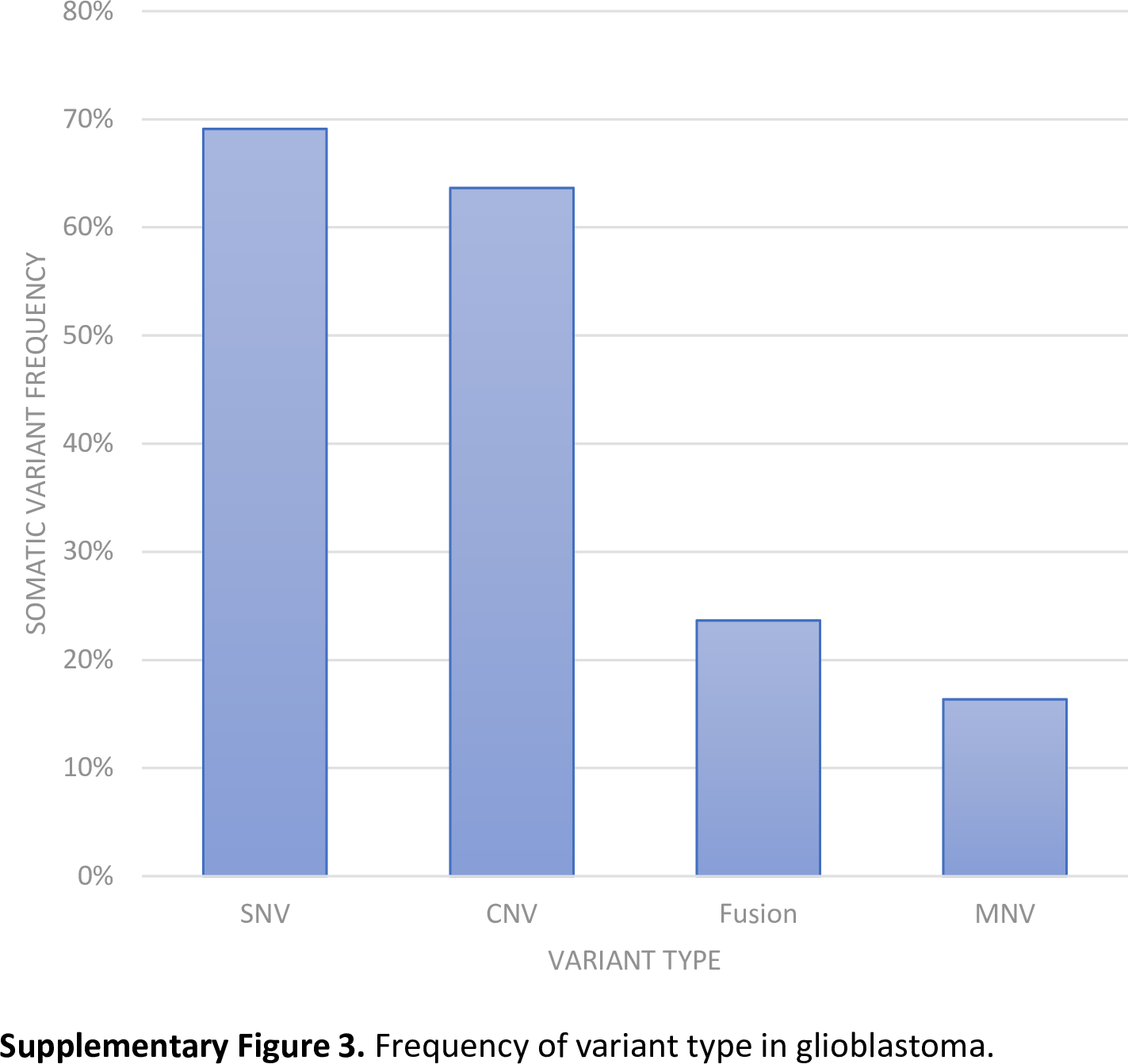

Supplement: S3 Fig — (TIF) [file pone.0245817.s011.tif]

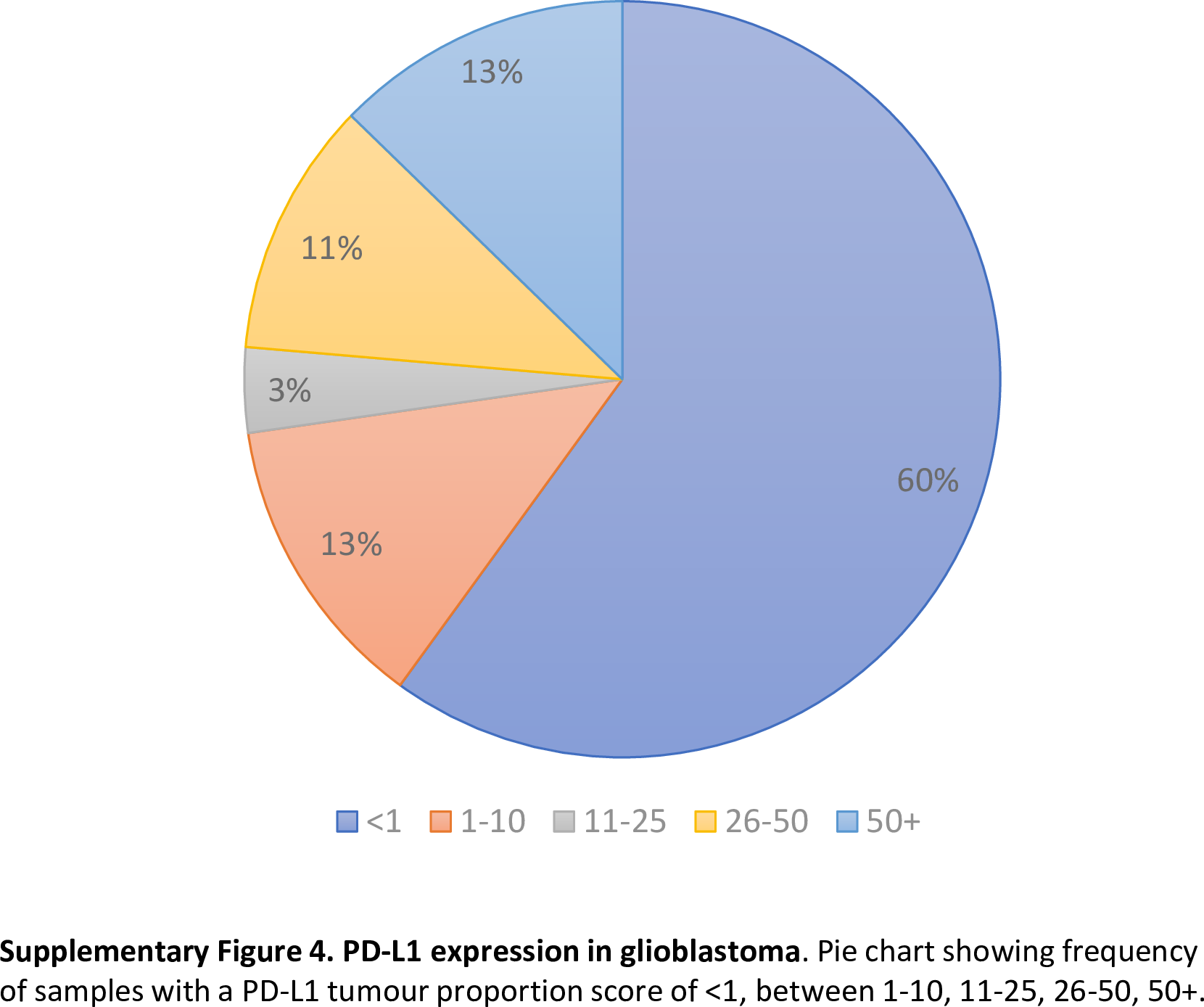

Supplement: S4 Fig — (TIF) [file pone.0245817.s012.tif]
