# Supplementary material for: Utilisation of semiconductor sequencing for the detection of predictive biomarkers in glioblastoma
Source: PLoS One. 2022 Mar 24;17(3):e0245817. doi: 10.1371/journal.pone.0245817 (PMC8947072; doi:10.1371/journal.pone.0245817)
Supplement: S3 Table — (PDF) [file pone.0245817.s003.pdf]

Supplementary Table 3. List of genes tested. Hotspot genes (n = 87), Full length (exon only) (n=48), CNV (n=43), Fusion drivers (n=51; highlighted in blue) partners (n=348). Note: Genes can be present in more than one category but only counted once in 505 gene total count.

Hotspot			
AKT1	FGFR4	MED12	SRC
AKT2	FLT3	MET	STAT3
AKT3	FOXL2	MTOR	TERT
ALK	GATA2	MYC	TOP1
AR	GNA11	MYCN	U2AF1
ARAF	GNAQ	MYD88	XPO1
AXL	GNAS	NFE2L2	
BRAF	H3F3A	NRAS	
BTK	HIST1H3B	NTRK1	
CBL	HNF1A	NTRK2	
CCND1	HRAS	NTRK3	
CDK4	IDH1	PDGFRA	
CDK6	IDH2	PDGFRB	
CHEK2	JAK1	PIK3CA	
CSF1R	JAK2	PIK3CB	
CTNNB1	JAK3	PPP2R1A	
DDR2	KDR	PTPN11	
EGFR	KIT	RAC1	
ERBB2	KNSTRN	RAF1	
ERBB3	KRAS	RET	
ERBB4	MAGOH	PHEB	
ERCC2	MAP2K1	RHOA	
ESR1	MAP2K2	ROS1	
EZH2	MAP2K4	SF3B1	
FGFR1	MAPK1	SMAD4	
FGFR2	MAX	SMO	
FGFR3	MDM4	SPOP	

Full length (exon only)	
ARID1A	PALB2
ATM	PIK3R1
ATR	PMS2
ATRX	POLE
BAP1	PTCH1
BRCA1	PTEN
BRCA2	RAD50
CDK12	RAD51
CDKN1B	RAD51B
CDKN2A	RAD51C
CDKN2B	RAD51D
CHEK1	RB1
CREBBP	RNF43
FANCA	SETD2
FANCD2	SLX4
FANCI	SMARCA4
FBXW7	SMARCB1
MLH1	STK11
MRE11A	TP53
MSH2	TSC1
MSH6	TSC2
NBN	
NF1	
NF2	
NOTCH1	
NOTCH2	
NOTCH3	

CNV	
AKT1	MDM2
AKT2	MDM4
AKT3	MET
ALK	MYC
AR	MYCL
AXL	MYCN
BRAF	NTRK1
CCND1	NTRK2
CCND2	NTRK3
CCND3	PDGFRA
CCNE1	PDGFRB
CDK2	PIK3CA
CDK4	PIK3CB
CDK6	PPARG
EGFR	RICTOR
ERBB2	TERT
ESR1	
FGF19	
FGF3	
FGFR1	
FGFR2	
FGFR3	
FGFR4	
FLT3	
IGF1R	
KIT	
KRAS	

Fusions															
A2M	CREB5	LSM12	RB1	ASIC2	ESR1	NACC2	SLMAP	BTAF1	GABBR2	OPHN1	TMPRSS2	CEL	KANK1	PPP4R3B	VOPP1
ABCB5	CTNNB1	LSM14A	RBMS3	ATAD2	ESRP1	NAV1	SND1	BTBD1	GATM	OXR1	TNIP1	CEP85L	KANK2	PRKACA	WASF2
ACACA	CUL1	LYN	RBPMS	ATAD5	ETV1	NCOA1	SNHG7	BTF3L4	GFPT1	PAPD7	TNKS2	CEP89	KCNQ5	PRKACB	WDR48
ACADM	CUX1	MACF1	RELA	ATF7IP	ETV4	NCOA4	SNX19	C11orf95	GHR	PAPSS1	TP53	CHD9	KCTD1	PRKAR1A	WHSC1L1
ACBD5	DAB2	MAD1L1	RET	ATG7	ETV5	NCOR2	SOX6	C7orf73	GIT2	PARK2	TP53BP1	CHTOP	KCTD7	PRKG2	WIPF2
ACTG2	DAB2IP	MBIP	RNF11	ATIC	ETV6	NDE1	SPAG9	C8orf34	GLIS3	PAX5	TPM1	CIC	KDELR2	PSMD11	YAP1
ADAM32	DCTN1	MCFD2	RNF130	ATP1B1	EZR	NF1	SPECC1	C9orf153	GNAI1	PAX8	TPM3	CIITA	KDM7A	PSPH	YTHDF3
ADAMTS1	DIP2C	MDM4	RNF213	ATRNL1	FAM114A	NFASC	SPECC1L	CAD	GNS	PCDHGA1	TPM4	CIT	KDR	PTEN	YWHAE
AES	DNAJB1	MEMO1	ROS1	AXL	FAM131B	NFIB	SPTBN1	CAND1	GOLGA4	PCM1	TPR	CLCN6	KIAA1468	PTPN3	ZC3HAV1
AFAP1	DTD1	MET	RP2	B4GALT1	FAM76A	NFKB2	SQSTM1	CAPRIN1	GOLGA5	PCNX	TRAF1	CLIP1	KIAA1549	PTPRK	ZCCHC8
AFF3	DYM	MGEA5	RSPO2	BAG4	FAT1	NIN	SRGAP3	CAPZA2	GOLGB1	PDE10A	TRAK1	CLIP2	KIAA1598	PTPRZ1	ZEB2
AGAP3	DYNC1I2	MIR143H	RSPO3	BAIAP2L1	FBXO28	NOL4	SSBP2	CARS	GOPC	PDE4DIP	TRIM24	CLIP4	KIF5B	PWWP2A	ZKSCAN1
AGBL4	DYNC2H1	MKRN1	RUFY2	BBS9	FCHSD1	NOTCH1	STK32B	CASP7	GRB7	PDE7A	TRIM27	CLTC	KLC1	QKI	ZKSCAN5
AGGF1	EBF1	MPRIP	SART3	BCAM	FGFR1	NOTCH4	STRN	CCAR2	GRHL2	PDGFRA	TRIM33	CNTLN	KLHL7	RABEP1	ZMYM2
AGK	EGFR	MRPL24	SCAF11	BCAN	FGFR1OP	NPC2	STRN3	CCDC127	GTF2I	PDGFRB	TRIM4	CNTRL	KRAS	RABGAP1L	ZMYND8
AGTRAP	EIF3E	MRPS33	SDC4	BCL2L11	FGFR1OP	NPM1	SUGCT	CCDC170	GTF2IRD1	PDHX	TRIO	COL14A1	KTN1	RAD18	ZNF226
AHCYL1	ELAVL3	MSN	SDCCAG3	BCR	FGFR2	NRG1	TACC1	CCDC6	GTF3C2	PDP1	TRIP11	COX5A	LMNA	RAD51B	ZNF703
AKAP12	EML4	MTHFD1L	SEC16A	BEND5	FGFR3	NSD1	TACC3	CCDC88A	HACL1	PDZRN3	TRMT61B	CPSF6	LRIG3	RAF1	ZSCAN30
AKAP13	EPHB2	MTMR12	SEC31A	BICC1	FGR	NTM	TANK	CCDC88C	HERPUD1	PIK3CA	TSEN2	CREB3L2	LRRFIP1	RANBP2	
AKAP9	EPS15	MYB	SEC61G	BICD2	FIP1L1	NTRK1	TAX1BP1	CCDC91	HIP1	PLAG1	TTLL7				
AKT2	ERBB2	MYBL1	SHROOM	BIN2	FKBP15	NTRK2	TBL1XR1	CCNY	HLA_A	PLIN3	TXLNA				
ALK	ERBB4	MYH13	SHTN1	BIRC6	FLT3	NTRK3	TENM4	CD44	HMGA2	POLH	TYK2				
AP3B1	ERC1	MYH9	SLC12A7	BRAF	FN1	NUB1	TERF2	CD74	HOMER1	PPARG	UBE2L3				
AR	ERG	MYO18A	SLC26A4	BRCA1	FNDC3B	NUDCD3	TERT	CDC27	HOOK3	PPFIBP1	UBN2				
ARHGEF2	ERLIN2	MYO5A	SLC34A2	BRCA2	FOXP1	NUP214	TFG	CDK5RAP2	IRF2BP2	PPHLN1	USP10				
ARMC10	ERP44	MYRIP	SLC3A2	BRD3	FXR1	NUTM1	TMEM106B	CDK6	JAK2	PPL	VAMP2				
ARMT1	ERVK3_1	MZT1	SLC45A3	BRD4	FYCO1	OFD1	TMEM178B	CDKN2A	JAKMIP1	PPM1G	VCL				
